# Phytochemical, pharmacological, pharmacokinetic and toxicological characteristics of *Ziziphi Spinosae Semen*: a review

**DOI:** 10.3389/fphar.2024.1504009

**Published:** 2024-11-29

**Authors:** Mei-Hua Liu, Hong-Xin Jin, Zhen Song, Jing-Yi Wang, De-Jiang Gao

**Affiliations:** ^1^ Research Center of Emotional Diseases, Shenyang Anning Hospital, Shenyang, China; ^2^ Shenyang Key Laboratory for Causes and Drug Discovery of Chronic Diseases, Shenyang Anning Hospital, Shenyang, China; ^3^ China Medical University the 4th People’s Hospital of Shen Yang, Shenyang, China

**Keywords:** *Ziziphi Spinosae Semen*, metabolite, pharmacokinetic, cardiovascular system, toxicology

## Abstract

*Ziziphi Spinosae Semen* (ZSS) is the seeds of *Ziziphus jujuba Mill. var. Spinosa* (Bunge) Hu ex H. F. Chou, which has the effects of nourishing heart and liver, tranquilizing heart and tranquilizing mind. With the development of research on the metabolites of ZSS, more than 160 metabolites have been isolated from ZSS, including saponins, alkaloids, flavonoids, fatty acids, volatile oils, polysaccharides and proteins. The active metabolites of ZSS have regulatory effects on the nervous system, cardiovascular system, hematopoietic system, immune system and substance metabolism, and have various pharmacological effects such as anti-oxidation, anti-aging and anti-cancer. Although many traditional uses of ZSS have been clarified, the relationship between its structure and function remains to be further studied. This article provides a review of the metabolites, pharmacological activity, pharmacokinetics and toxicology of ZSS, and explores the future research prospects and existing problems of ZSS, so as to provide reference for further research and establishment of quality control standards of ZSS.

## 1 Introduction

**FIGURE 1:**
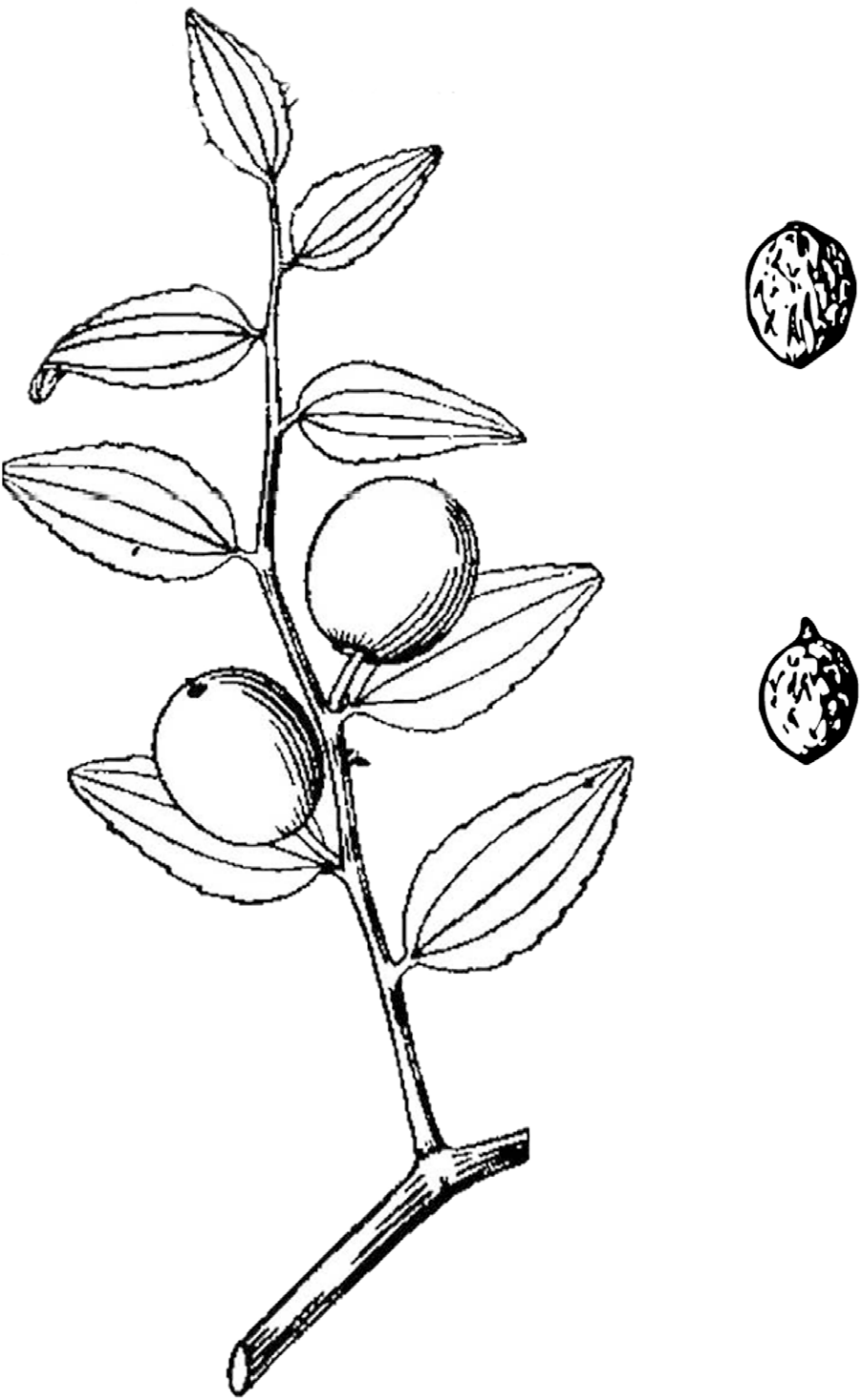
Plant diagram of *Ziziphi Spinosae Semen* (Chinese Flora Editing Committee, 1993).

*Ziziphi Spinosae Semen* (ZSS) is a primary choice in traditional Chinese medicine for the clinical treatment of insomnia. It has a history of over 2,000 years of use in China and is recognized as one of the first substances classified as ‘medicine and food homologous’ by China. ZSS is widely distributed across Asia, Europe, and Australia, particularly in northwest China and the Yellow River Basin. It is derived from the dried mature seeds of *Ziziphus jujuba Mill. var. Spinosa* (Bunge) Hu ex H. F. Chou (Gao et al., 2013) (Figure 1).

ZSS can be utilized either alone or in conjunction with other medicinal substances. Currently, over 30 ZSS prescriptions are documented in the Pharmacopoeia of the People’s Republic of China (ChP), primarily for the treatment of palpitations, insomnia, dizziness, depression, anxiety, and various other conditions. To date, researchers have isolated and identified more than 160 metabolites from ZSS, including saponins, alkaloids, flavonoids, fatty acids, volatile oils, polysaccharides, and several inorganic compounds (Yin et al., 1997; Liu et al., 2016; Javier and Laura, 2017; Guo et al., 2021; Wang et al., 2022). Pharmacological studies have demonstrated that the primary pharmacological activities of ZSS are sedation and hypnosis. With advancements in research technology, the current focus has expanded to include areas such as enhancing learning and memory, antioxidant effects, anti-anxiety, anti-depression, anti-inflammatory properties, cardiovascular protection, blood pressure reduction, lipid-lowering, anti-aging, and anti-tumor effects (Zeng et al., 2012; Jiang et al., 2007; Chen et al., 2023).

In this review, we searched the relevant literature, Ph.D. and MSc dissertations of *Ziziphi Spinosae Semen* from PubMed, Web of Science, Baidu Scholar, CNKI and other scientific databases, and summarized the characteristics of phytochemistry, pharmacology, pharmacokinetics and toxicology of ZSS. Finally, this paper also discusses the existing problems and research directions of ZSS.

## 2 Metabolites

The chemical composition of ZSS is complex and diverse. To date, more than 160 metabolites have been isolated and identified from ZSS, primarily including saponins, flavonoids, triterpenoids, alkaloids, fatty oils, steroids, phenolic acids, amino acids, and trace elements.

### 2.1 Saponins

**FIGURE 2:**
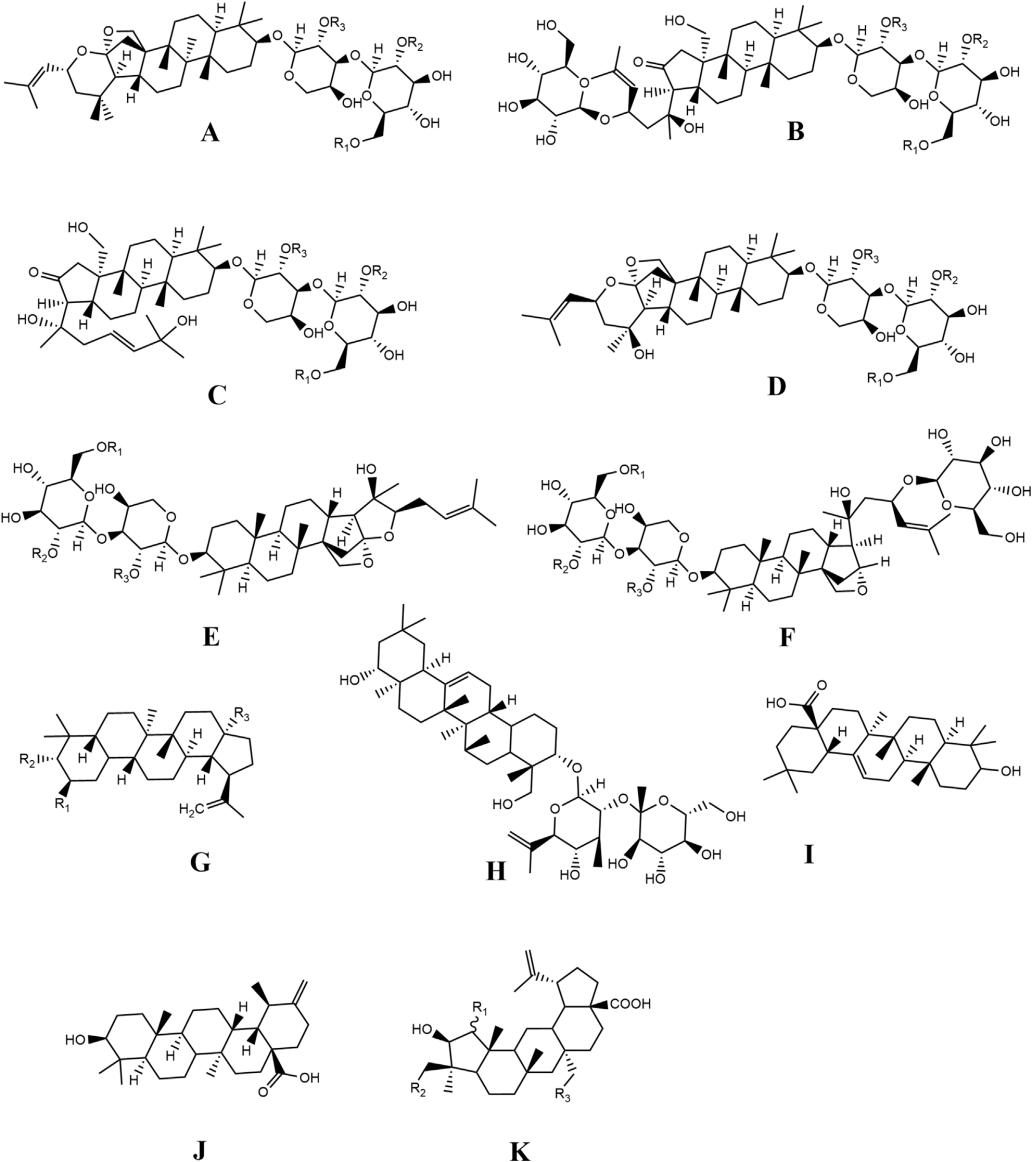
Chemical structures of the saponins in ZSS (1-33).

Jujubosides are widely recognized sedative and hypnotic substances with high content in ZSS. Among these, dammarane-type triterpenoid saponins are the main metabolites (Yin et al., 1997). In 1978, jujuboside A, B and C were first extracted from the methanol extract of ZSS, which were similar to the active ingredient saponins in *Panax notoginseng* (Burkill) F. H. Chen ex C. H. Chow and *Panax ginseng* C. A. Mey. It is the most reported metabolite (Otsuka et al., 1978). The mass fraction of total saponins in ZSS was 0.0916%, and sapogenins included tetracyclic triterpenoids and pentacyclic triterpenoids. Wang et al. isolated for the first time metabolites of lupine type, namely, lupinol and methyl betulinate from the ZSS. In subsequent studies, they also discovered metabolites of lupine type contained in ZSS, such as Betulin, Betulic acid, and Alphitolic acid (Wang et al., 2008). At present, more than 30 saponins have been identified (Table 1; Figure 2).

**TABLE 1:** Saponins isolated from ZSS.

No.	Metabolites	Core	Substituent			References
			R1	R2	R3	
1	Jujuboside A	A	glc	xyl	rha	Otsuka et al. (1978)
2	Jujuboside A1	A	glc	xyl	fuc	Matsuda et al. (1999), Liu et al. (2004)
3	Jujuboside A2	A	glc	xyl	6-deoxy-talose	Liu et al. (2004)
4	Jujuboside B	A	H	xyl	rha	Otsuka et al. (1978)
5	Jujuboside B1	A	H	xyl	fuc	Matsuda et al. (1999)
6	Jujuboside C	A	glc	glc	rha	Matsuda et al. (1999)
7	Acetyljujuboside B	A	Ac	xyl	rha	Matsuda et al. (1999)
8	Zizyphus saponin II	A	H	H	rha	Wang et al. (2013)
9	Zizyphus saponin III	A	H	xyl	6-deoxy-talose	Liu (2013)
10	Jujuboside I	A	H	glc	rha	Wang et al. (2013)
11	Protojujuboside A	B	glc	xyl	rha	Matsuda et al. (1999)
12	Protojujuboside B	B	H	xyl	rha	Matsuda et al. (1999)
13	Protojujuboside B1	B	H	xyl	fuc	Matsuda et al. (1999)
14	Jujuboside H	C	glc	xyl	rha	Wang et al. (2009a)
15	Jujuboside G	C	H	xyl	rha	Wang and Yang (2008)
16	Jujuboside II	D	H	xyl	rha	Wang et al. (2013)
17	Jujuboside III	E	H	xyl	rha	Wang et al. (2013)
18	Jujuboside IV	E	glc	xyl	rha	Wang et al. (2013)
19	Jujuboside E	F	glc	xyl	rha	Bai et al. (2003)
20	Betulonic acid	G	H	OH	COOH	Zhang et al. (2016)
21	Alphitolic aicd methyl ester	G	OH	OH	COOCH_3_	He et al. (2006)
22	Lupeol	G	H	OH	CH_3_	Wang and Yang (2008)
23	Methyl betulinate	G	H	OH	COOCH_3_	Wang and Yang (2008)
24	Betulin	G	H	OH	CH_2_OH	Zeng et al. (1986)
25	Alphitolic acid	G	OH	OH	COOH	Zhang et al. (2016)
26	Betulinic acid	G	H	OH	COOH	Zeng et al. (1986)
27	Azukisaponin II	H	—	—	—	Li et al. (2005)
28	Oleanolic acid	I	—	—	—	Cao and Wang (2009)
29	Zizyphursolic acid	J	—	—	—	Yang et al. (2013)
30	24-Hydroxyceanothic acid	K	αCOOH	OH	H	Zhang et al. (2016)
31	27-Hydroxyceanothic acid	K	αCOOH	H	OH	Zhang et al. (2016)
32	Ceanothic acid	K	αCOOH	H	H	Zeng et al. (1986)
33	Epiceanothic acid	K	βCOOH	H	H	Zhang et al. (2016)

### 2.2 Alkaloids

**FIGURE 3:**
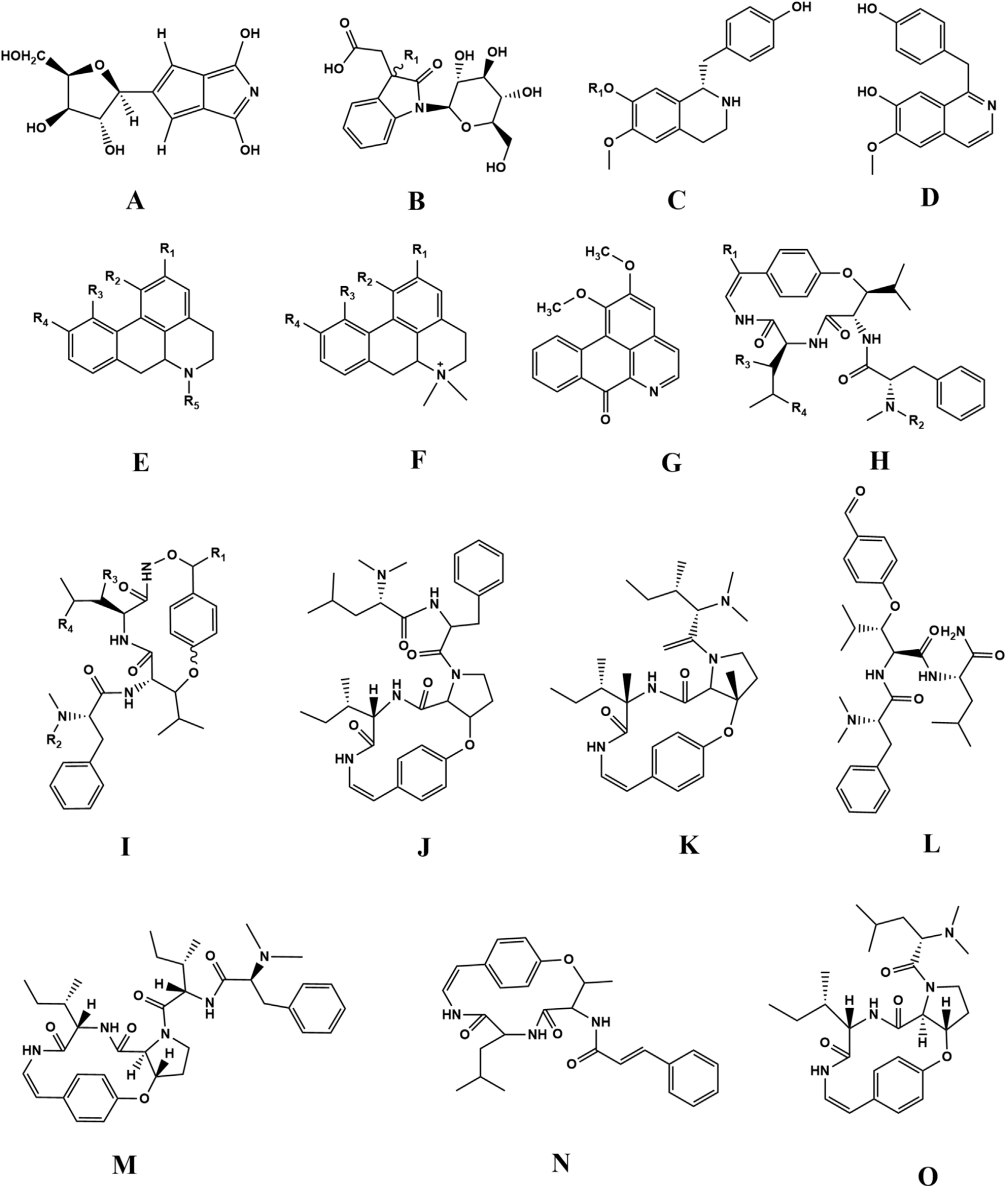
Chemical structures of the alkaloids in ZSS (34-59).

There are relatively few studies on the separation of alkaloids in ZSS (Liu et al., 2004). The first alkaloids isolated from ZSS were lysicamine and juzirine (Sun et al., 2013). As research has progressed, more than 20 alkaloids have been isolated and identified from ZSS (Table 2; Figure 3). The alkaloids in ZSS can be categorized into two main groups: the first group includes cyclic peptide alkaloids such as sanjoinine A, sanjoinine B, sanjoinine D, sanjoinine F, and sanjoinine G1 (Yin et al., 1997), while the second group consists of aporphine alkaloids, including nuciferine, coclaurine, nornuciferine, and norisocorydine. Additionally, ZSS contains other alkaloids such as magnoflorin, zizyphusine, and N-methylasimilobine (Liu et al., 2016).

**TABLE 2:** Alkaloid isolated from ZSS.

No.	Metabolites	Core	Substituent					References
			R1	R2	R3	R4	R5	
34	6-(2′,3′-dihydroxy-4′-hydroxymethyl-tetrahydro-furan-1′-yl)-Cyclopentene [C] pyrrole-1,3-diol	A	—	—	—	—	—	Xie et al. (2011)
35	3*S*-1-*N*-β-*D*-Glucopyranosyl-2-oxo-3-hydroxy-indole-3-acetic acid	B	OH(S)	—	—	—	—	Li et al. (2014)
36	3*R*-1-*N*-β-*D*-Glucopyranosyl-2-oxo-3-hydroxy-indole-3-acetic acid	B	OH(R)	—	—	—	—	Xie et al. (2011)
37	Coclaurine	C	—	—	—	—	—	Mesmar et al. (2022)
38	Juzirine	D	—	—	—	—	—	Yin et al. (1997)
39	Norisocorydine	E	OCH_3_	OCH_3_	OH	OCH_3_	H	Han et al. (1989)
40	Caaverine	E	OCH_3_	OH	H	H	H	Mesmar et al. (2022)
41	Asimilobine	E	OH	OCH_3_	H	H	H	Yin et al. (1997)
42	Nornuciferine	E	OCH_3_	OCH_3_	H	H	H	Mesmar et al. (2022)
43	*N*-Methylasimilobine	E	OH	OCH_3_	H	H	CH_3_	Han et al. (1989)
44	Nuciferine	E	OCH_3_	OCH_3_	H	H	CH_3_	Han et al. (1989)
45	Zizyphusine	F	OH	OH	OCH_3_	OCH_3_	—	Mesmar et al. (2022)
46	Magnoflorine	F	OCH_3_	OH	OH	OCH_3_	—	Lee et al. (2012)
47	Lysicamine	G	—	—	—	—	—	Yin et al. (1997)
48	6-Glc-coclaurine	C	Glu	—	—	—	—	Zhang et al. (2016)
49	Sanjoinine A	H	H	CH_3_	H	CH_3_	—	Mesmar et al. (2022)
50	Sanjoinine F	H	H	CH_3_	OH	CH_3_	—	Mesmar et al. (2022)
51	Sanjoinine B	H	H	H	H	CH_3_	—	Mesmar et al. (2022)
52	Sanjoinine D	I	H	CH_3_	CH_3_	H	—	Mesmar et al. (2022)
53	Sanjoinine G1	I	OCH_3_	CH_3_	H	CH_3_	—	Xie et al. (2011)
54	Lotusine B	J	—	—	—	—	—	Yin et al. (1997)
55	Ramosine A	K	—	—	—	—	—	Yin et al. (1997)
56	Anjoinine G2	L	—	—	—	—	—	Mesmar et al. (2022)
57	Amphibine D	M	—	—	—	—	—	Han et al. (1989)
58	Sanjoinenine	N	—	—	—	—	—	Han et al. (1989)
59	Mucronine J	O	—	—	—	—	—	Mesmar et al. (2022)

### 2.3 Flavonoids

**FIGURE 4:**
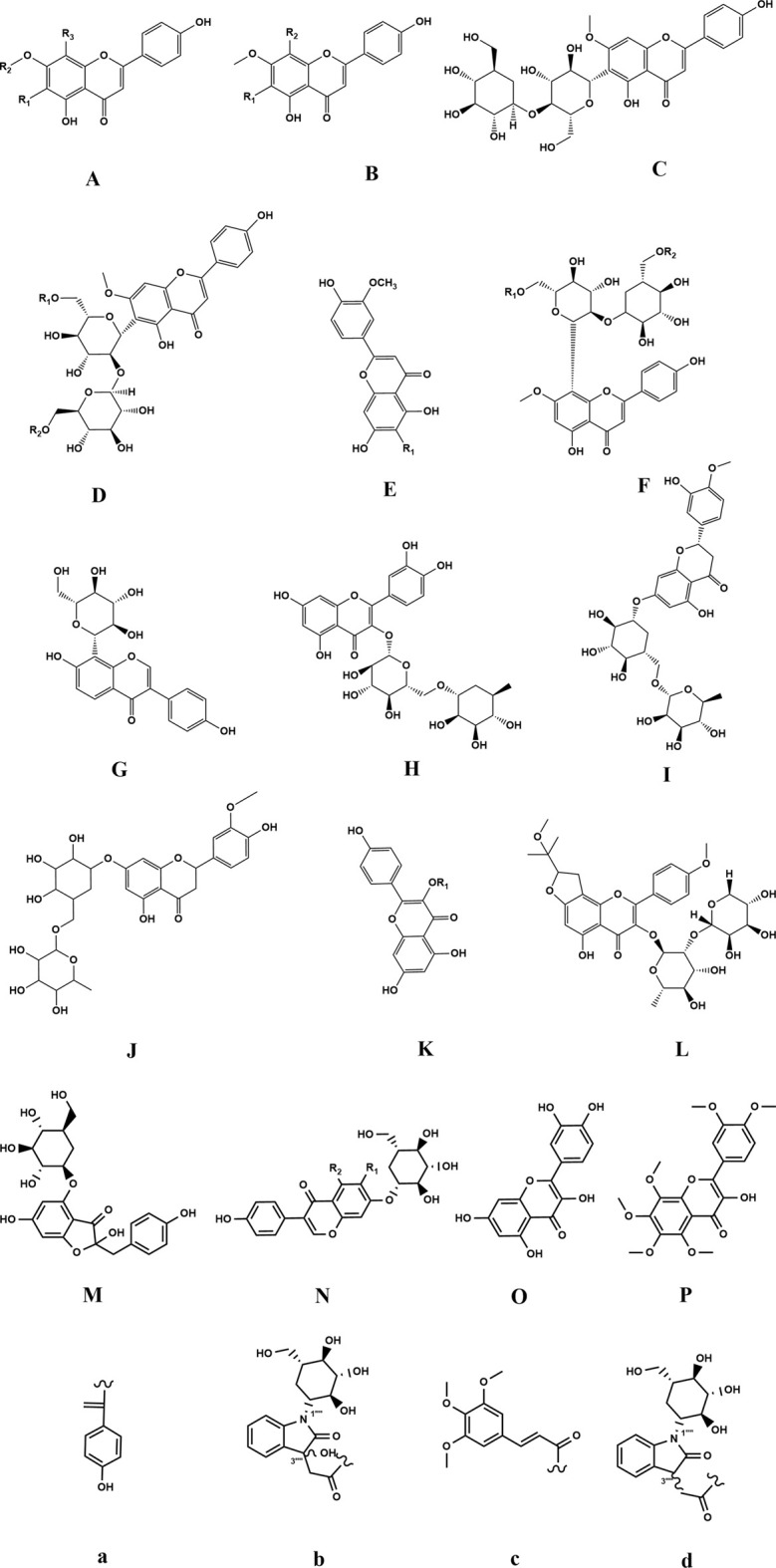
Chemical structures of the flavonoids in ZSS (60-109).

Another significant bioactive metabolite in ZSS is flavonoids, particularly represented by spinosin, which has a mass fraction of 0.95%. Its structure primarily consists of Flavone C-glycosides. In the late 1970s, four C-6 flavonoid C-glycosides with genkwanin as the parent nucleus were isolated from the methanol extract of ZSS for the first time, namely, spinosin and its acylated derivatives (Woo et al., 1979; Woo et al., 1980). With advancements in the determination of the structures of flavonoid glycosides, an increasing number of flavonoid metabolites have been isolated and identified from ZSS, including swertisin, 6‴-p-hydroxybenzoylspinosin, 6‴-feruloylspinosin, 6‴-sinapoylspinosin, six-p-coumaroylspinosin, and puerarin (Cheng et al., 2000; Shin et al., 1982; Xie et al., 2011). Wang et al. isolated a new angular furan flavonoid rhamnoside from ZSS, named Spinorhamnoside (Wang et al., 2005). Zhang et al. obtained two new flavonoid glycosides, 6‴-dihydrophaseoylspinosin and 6″, 6‴-diferuloylspinosin, from the methanol extract of ZSS (Zhang et al., 2016). To date, more than 40 flavonoids have been isolated and identified from ZSS (Table 3; Figure 4).

**TABLE 3:** Flavonoids isolated from ZSS.

No.	Metabolites	Core	Substituent			References
			R1	R2	R3	
60	Apigenin-6-C-glucopyranoside	A	Glc	H	H	Cheng et al. (2000)
61	Isovitexin-2″-O-β-D-glucopyranoside	A	Glc-glc	H	H	Cheng et al. (2000)
62	Isovitexin-2″-O-(6-p-coumaloyl)-glucopyranoside	A	Glc-glc-*p*-coumaroyl	H	H	Zhang et al. (2016)
63	Isovitexin-2″-O-(6-feruloyl)-glucopyranoside	A	Glc-glc-feruloyl	H	H	Zhang et al. (2016)
64	Vitexin	A	H	H	Glc	Cheng et al. (2000)
65	Vicenin II	A	gGlc	H	Glc	Zhang et al. (2016)
66	Swertisin	B	Glc	H	—	Xie et al. (2011)
67	Zivulgarin	C	—	—	—	Wu et al. (2011)
68	Spinosin	D	H	H	—	Woo et al. (1979)
69	6‴-Vanilloylspinosin	D	H	vanilloy	—	Wu et al. (2011)
70	6‴-(4′′′′-O-β-D-glucopyranosyl)-Vanilloylspinosin	D	H	Vanilloy-glc	—	Xie et al. (2011)
71	6‴-Dihydrophaseoylspinosin	D	H	dihydrophaseoyl	—	Zhang et al. (2012)
72	6‴-Sinapoylspinosin	D	H	sinapoyl	—	Woo et al. (1980)
73	6‴-p-Coumaloylspinosin	D	H	P-coumaroyl	—	Woo et al. (1980)
74	6‴-Feruloylspinosin	D	H	feruloyl	—	Woo et al. (1980)
75	6″,6‴-Diferuloylspinosin	D	feruloyl	feruloyl	—	Fu et al. (2017)
76	6″-O-Feruloylspinosin	D	feruloyl	H	—	Fu et al. (2017)
77	6″-O-Feruloyl-6‴-p-hydroxybenzoylspinosin	D	feruloyl	a	—	Fu et al. (2017)
78	6‴-O-(3S-1-N-β-D-glucopyranosyl-2-oxo-3-hydroxy-indole-3-acetyl) Spinosin	D	H	b (3””-S)	—	Li et al. (2014)
79	6‴-O-(3R-1-N-β-D-glucopyranosyl-2-oxo-3-hydroxy-indole-3-acetyl) Spinosin	D	H	b (3””-R)	—	Li et al. (2014)
80	6″-O-(3S-1-N-β-D-glucopyranosyl-2-oxo-3-hydroxy-indole-3-acetyl) Spinosin	D	b (3””-S)	H	—	Li et al. (2014)
81	6″-O-(3R-1-N-β-D-glucopyranosyl-2-oxo-3-hydroxy-indole-3-acetyl) Spinosin	D	b (3””-R)	H	—	Li et al. (2014)
82	6″-O-(3S-1-N-β-D-glucopyranosyl-2-oxo-3-hydroxy-indole-3-acetyl)-6‴-Feruloylspinosin	D	b (3””-S)	feruloyl	—	Li et al. (2014)
83	6″-O-(3R-1-N-β-D-glucopyranosyl-2-oxo-3-hydroxy-indole-3-acetyl)-6‴-Feruloylspinosin	D	b (3””-R)	feruloyl	—	Li et al. (2014)
84	6‴-p-Hydroxybenzoylspinosin	D	H	P-hydroxybenzoyl	—	Wu et al. (2015)
85	6‴-(3′′′′,4′′′′,5′′′′-trimethoxyl)-(E)-Cinnamoylspinosin	D	H	c	—	Zhang et al. (2016)
86	6‴-(−)-Phaseoylspinosin	D	H	phaseoyl	—	Zhang et al. (2016)
87	6‴-(4′′′′-O-β-D-glucopyranosyl)-Benzoylspinosin	D	H	4-O-glc-benzoyl	—	Zhang et al. (2016)
88	6″-Feruloyl-6‴-vanillyolspinosin	D	feruloyl	vanilloy	—	Chen et al. (2015)
89	6‴-(N-β-D-glucopyranosyl)-2′′′′,3′′′′-Dihydro-2′′′′-oxo-3′′′′-yl-acetate spinosin	D	H	d (3””-S)	—	Wu et al. (2015)
90	Epi-6‴-(N-β-D-glucopyranosyl)-2′′′′,3′′′′-dihydro-2′′′′-oxo-3′′′′-yl-acetate spinosin	D	H	d (3””-R)	—	Wu et al. (2015)
91	Isoscoparin-2″-O-(6-feruloyl)-glucopyranoside	E	Glc-glc-feruloyl	—	—	Zhang et al. (2016)
92	Isospinosin	F	H	H	—	Cheng et al. (2000)
93	6‴-Feruloylisospinosin	F	H	feruloyl	—	Cheng et al. (2000)
94	6″,6‴-Diferuloylisospinosin	F	feruloyl	feruloyl	—	Zhang et al. (2016)
95	Isowertisin	B	H	Glc	—	Wang et al. (2008)
96	Puerarin	G	—	—	—	Cheng et al. (2000)
97	Naringin	A	H	Glc-rha	H	Zhang et al. (2015)
98	Rutin	H	—	—	—	Zhang et al. (2015)
99	Hesperidin	I	—	—	—	Xie et al. (2011)
100	Clematine	J	—	—	—	Xie et al. (2011)
101	Nicotiflorin	K	Glc-rha	—	—	Zhang et al. (2016)
102	Camelliaside B	K	Glc-rha (6″)-xyl (2″)	—	—	Li et al. (2005)
103	Spinorhamnoside	L	—	—	—	Lee et al. (2012)
104	Hovetrichoside C	M	—	—	—	Li et al. (2005)
105	Glycitin	N	OCH_3_	H	—	Wang et al. (2005)
106	Genistin	N	H	OH	—	Wang et al. (2005)
107	Quercetin	O	—	—	—	Cao and Wang (2009)
108	5,6,7,8,3′,4′-Hexamethoxy	P	—	—	—	Wang et al. (2005)
109	Apigenin	A	H	H	H	Zhang et al. (2015)

### 2.4 Fatty acids and volatile oils

**FIGURE 5:**
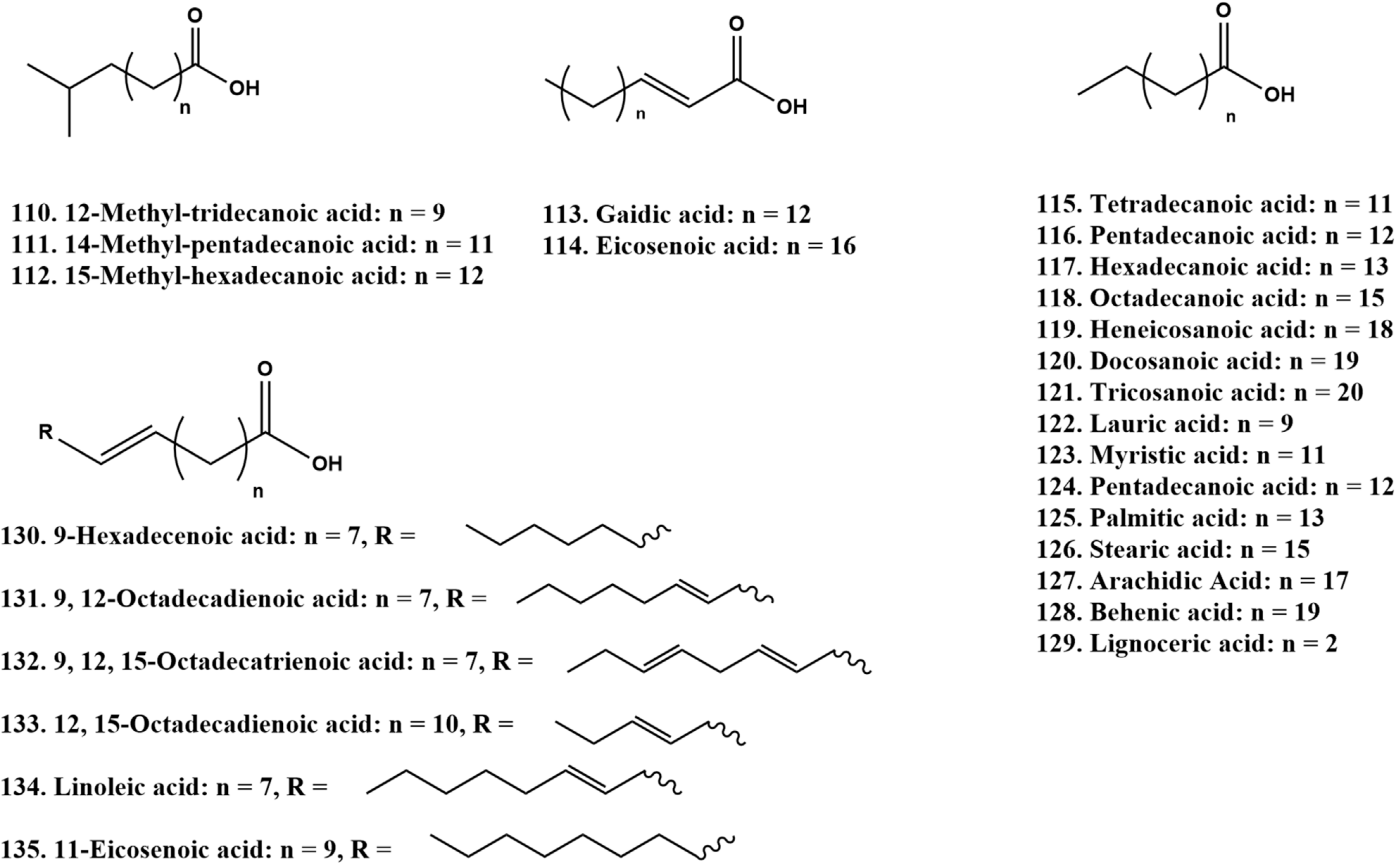
Chemical structures of the fatty acids and volatile oils in ZSS (110-135).

The fatty acid content in ZSS was as high as 32%, comprising 17 different fatty acids (Figure 5). Among these, unsaturated fatty acids accounted for 80.20% of the total fatty acids, including arachidic acid, myristic acid, lauric acid, palmitic acid, stearic acid, linoleic acid, pentadecanoic acid, hexadecenoic acid, and docosanoic acid (Chen et al., 2001). In 2001, supercritical fluid extraction technology was employed for the first time to extract oils from ZSS, resulting in the identification of 12 types of fatty acids. Notably, linoleic acid constituted 37.14% of the total, while oleic acid made up 38.73%. Lu et al. demonstrated that the fatty oils of ZSS were primarily composed of six fatty acids, with oleic acid, linoleic acid, and linolenic acid being the most abundant, at 49.1%, 26.0%, and 4.1%, respectively (Lu et al., 2006). In addition, the oil of ZSS also contains a small amount of lauric acid, palmitoleic acid, and docosahexaenoic acid (Li et al., 1993).

### 2.5 Other constituents

**FIGURE 6:**
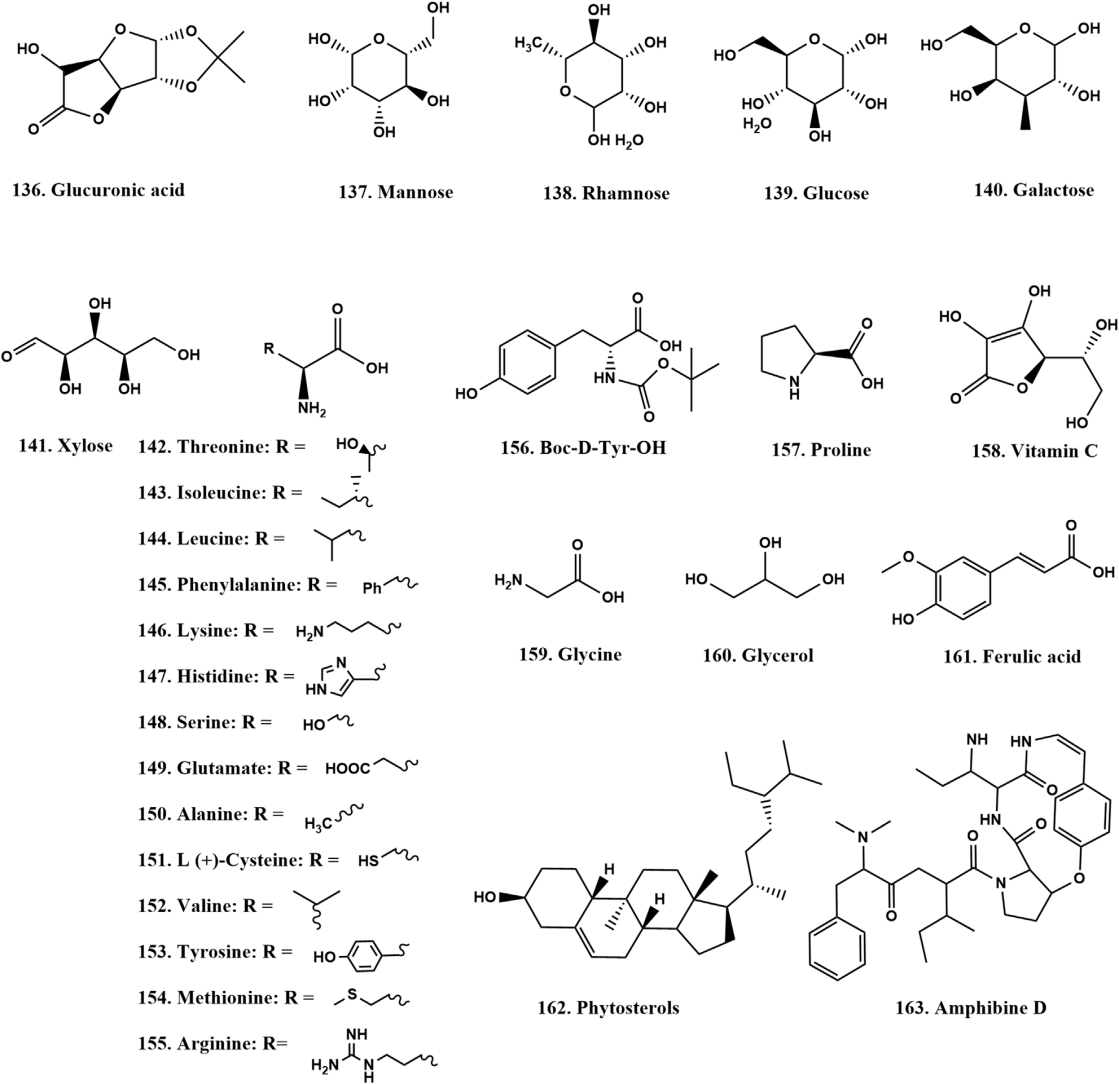
Chemical structures of other compounds in ZSS (136-163).

ZSS contains polysaccharides (Liu et al., 2020), phytol, phenolic acid (Wang et al., 2008; Guo et al., 1995), vitamin C, and a significant amount of cyclic adenosine monophosphate (cAMP) (Pandey et al., 1984). Additionally, ZSS provides seven essential trace elements, including iron (Fe), manganese (Mn), zinc (Zn), and selenium (Se), as well as eight essential amino acids, such as tyrosine, methionine, valine, and threonine (Figure 6) (Zhang et al., 1997; Dong et al., 1993).

## 3 Pharmacology

Pharmacological studies have shown that ZSS has a certain regulatory effect on the central nervous system and cardiovascular system. ZSS mainly treats restlessness, palpitation, insomnia and dreaminess. Modern pharmacological studies have found that ZSS also has sedative, hypnotic, lipid-lowering, improve myocardial ischemia, lipid-lowering and improve immunity and so on.

### 3.1 Effects on the nervous system

#### 3.1.1 Sedative hypnotic effect

ZSS is one of the classic traditional Chinese medicine for the treatment of anxiety and insomnia. Contemporary pharmacological research has identified saponins, flavonoids, alkaloids, and fatty acids as the primary bioactive metabolites that contribute to the sedative and hypnotic properties of ZSS. Raw ZSS (5.46 g/kg) and roasted ZSS (2.73 g/kg) demonstrated sedative and hypnotic properties by elevating the levels of nitric oxide (NO) in the hypothalamus and enhancing the activity of nitric oxide synthase (NOS). This led to a decrease in the duration of wakefulness and an extension of the slow-wave sleep phase in insomnia rats. Furthermore, the administration of 10.9 g/kg of roasted ZSS significantly prolonged sleep duration in mice subjected to pentobarbital sodium, exhibiting effects comparable to those of the established anxiolytic agent, diazepam (Zhai et al., 2015).

Flavonoids, polysaccharides, and saponins are the main active metabolites in ZSS. Mice were administered physical solutions of flavonoids, saponins, and polysaccharides at doses of 17 g/kg via gavage. The walking time and coordination ability of the mice in the flavonoid and saponin groups were significantly reduced, while the sleep duration was significantly prolonged, demonstrating notable sedative and hypnotic effects (Jiang et al., 2007). However, this study did not further explore the underlying mechanisms of these effects. Administration of 10 mg/kg of saponins has been demonstrated to effectively alleviate insomnia induced by chlorpheniramine through the activation of the cAMP/CREB/BDNF and PI3K/Akt signaling pathways. This intervention resulted in a significant reduction in the total distance traveled during the open field test in rats, an increase in resting time, and enhanced performance in the water maze test, as evidenced by increased time spent in the target quadrant, higher average speed, and a greater number of entries. Furthermore, the sedative and hypnotic effects of this dosage surpass those observed in the high-dose group (20 mg/kg), indicating a nonlinear dose-response relationship (Heng et al., 2022).

#### 3.1.2 Antidepressant effects

In recent years, through animal experiments and clinical studies, it has been proven that ZSS is one of the commonly used traditional Chinese medicines for antidepressant effects. ZSS could regulate the gut microbiota structure in rats subjected to chronic unpredictable mild stress, thereby alleviating excessive inflammatory responses and tryptophan metabolic imbalances, exerting its antidepressant effects (Du et al., 2024). The research investigated the antidepressant properties of alkaloids and saponins derived from ZSS in a murine model of depression. The findings indicated that the combination of alkaloids (15 mg/kg) and saponins (110 mg/kg) increased the levels of norepinephrine (NE), dopamine (DA), and serotonin (5-HT) in the hippocampus and prefrontal cortex of depressed mice by 14.65%, 87.72%, 33.44%, 25.64%, 25.39%, and 70.78%, respectively, effectively alleviating the depression in the mice (Li et al., 2022).

#### 3.1.3 Anxiolytic effect

The research examined the impact of methanol extracts derived from ZSS (MESZS) on anxiety associated with ethanol withdrawal (EtOHW). The findings revealed that EtOHW led to an upregulation of corticotropin-releasing factor (CRF) and its receptor (CRFR1) at both the gene and protein levels within the central nucleus of the amygdala (CeA). This upregulation was effectively mitigated by the administration of 180 mg/kg of MESZS. Furthermore, EtOHW was found to increase the expression of nociceptin/orphanin FQ peptide (NOP) mRNA and protein in the amygdala, while the levels of N/OFQ mRNA remained stable; notably, the administration of 180 mg/kg of MESZS further augmented these increases. Additionally, the introduction of CRF or the selective NOP antagonist UFP-101 into the CeA subsequent to MESZS treatment negated the anticipated anxiolytic effects of the 180 mg/kg/d dosage of MESZS. These results suggested that MESZS alleviates anxiety related to EtOHW by enhancing the signaling pathways of CRF/CRFR1 and N/OFQ/NOP within the CeA (Li et al., 2019).

GABAA receptor is generally considered to be the target of sedative hypnotic drugs. Spinosin, a primary flavonoid in ZSS, has been shown to significantly increase the time ratio and frequency ratio of mice entering the open arm in the EPM, light-dark box experiment, and open field test, without affecting spontaneous activity, thus exhibiting a consistent anxiolytic-like effect. Investigations have indicated that the anxiolytic effect of spinosin may be mediated via the GABAergic and serotonergic systems (Liu et al., 2015).

#### 3.1.4 Learning and memory improvements

Currently, there are few reports on the impact of ZSS on learning and memory abilities. Water soluble extract from ZSS (WSE) significantly reduced the number of errors (NOE) in the Y-maze and passive avoidance tests in mice with alcohol-induced memory retrieval impairment, increased the transfer latency time (TLT) and electrical stimulation time (EST). The moderate dose (200 mg/kg) and high dose (400 mg/kg) of WSE showed stronger effects compared to the 1,520 mg/kg dose of piracetam (*p* < 0.01). This study preliminarily confirmed that Spinosin, JuA, and JuB may be the main factors determining activity (Zhang et al., 2014), but how to control the dose of ZSS and the related mechanisms of action in terms of cognitive enhancement still need further exploration.

The effects of intraventricular injection of luA (0.02 and 0.2 mg/kg) on Aβ1-42-induced cognitive impairment were evaluated. The results showed that JuA intraventricular treatment significantly alleviated learning and memory impairments induced by Aβ1-42 in mice, reduced the levels of Aβ1-42 in the hippocampus, inhibited the activity of acetylcholinesterase (AChE) and nitric oxide (NO), and decreased the content of malondialdehyde (MDA) in the hippocampus and cerebral cortex of cognitively impaired mice. Indicated that JuA may serve as a potential therapeutic agent for treating Alzheimer’s disease (Liu et al., 2014).

#### 3.1.5 Anti-stress ulcer effect

The formation of stress ulcer is related to the dysfunction of the central nervous system. ZSS could improve stress ulcer by balancing the process of central excitation and inhibition. The ulcer index and ulcer inhibition percentage of mice were detected by inverted ulcer model. Research was conducted on the impact of ZSS on stress ulcers in mice. ZSS (2,500 mg/kg) had a significant inhibitory effect on stress-induced ulcers. Currently, there is limited research on the role of ZSS in this context, and no studies have explored its underlying mechanisms. Investigating the mechanisms by which ZSS treats stress ulcers is of considerable importance (Li and Zheng, 2003) (Table 4).

**TABLE 4:** Effects of ZSS on the nervous system.

Pharmacological effects	Extracts/Metabolites	Model	Dose range tested	Mechanism	Ref.
Sedative hypnotic effect	SZJ-I and SZJ-II	Male ICR mice	20, 40 and 80 mg/kg	The expression of 5-hydroxytryptamine 1A (5-HT1A), 5-hydroxytryptamine 2A (5-HT2A), GABAA receptor α2 (GABAARα2), GABAA receptor α3 (GABAARα3), glutamate decarboxylase (GAD) 65/67, IL-6 and IL-1β in hypothalamus and hippocampus were regulated	Shen et al. (2020)
	Jujuboside	Male ICR mice	9 mg/kg	Regulating 5-hydroxytryptamine energy system	Cao et al. (2010)
	Extract of ZSS	SD rats	13.50, 9.01 and 4.50 mg/kg	Regulating the expression levels of GABA receptor subunit alpha-1 (GABAARα1) and GABA acid receptor subunit gamma-2 (GABAARγ2) receptors in the hypothalamus and hippocampus tissue sections	Xiao et al. (2022)
	Total saponins	SD rats	10、20 g/kg	Enhance sleep through the 5-hydroxytryptamine mechanism	Zhai et al. (2015)
Antidepressant effect	Extract of ZSS	Rats	--	Regulate the level of pyroglutamic acid to resist depression	Du et al. (2024)
	Alkaloids	Male ICR mice	15 mg/kg	The contents of norepinephrine (NE), dopamine (DA) and serotonin (5-HT) were upregulated	Li et al. (2022)
	Saponins	Male ICR mice	110 mg/kg	The contents of norepinephrine (NE), dopamine (DA) and serotonin (5-HT) were upregulated	Li et al. (2022)
Anxiolytic effect	Ethanol extract of ZS	SD rats	60, 180 mg/kg	Improving the transmission of CRF/CRFR1 and N/OFQ/NOP in CeA	Li et al. (2019)
	Spinosin	Mice	2.5, 5 mg/kg	Regulates GABAA and 5-HT1A receptors	Liu et al. (2015)
	Jujuboside A	Male Kunming mice	0.02, 0.2 mg/kg	Aβ1-42-induced histopathological damage was antagonized, the activity of caspase-9 and caspase-3 in mitochondria was decreased, and apoptosis was reduced	Liu et al. (2014)
Anti-stress ulcer effect	ZSS extract	Kunming mice	2.5, 10 and 25 g/kg	The process of balancing central excitation and inhibition	Li and Zheng (2003)

### 3.2 Effects on cardiovascular system

ZSS is significant in the context of cardiovascular system diseases. It contains a notable quantity of potassium, which contributes to the maintenance of electrolyte balance and may aid in the reduction of hypertension. Furthermore, the polyphenolic metabolites present in ZSS have been shown to effectively lower levels of low-density lipoprotein in the bloodstream, thereby diminishing the risk of cardiovascular disease (Table 5).

**TABLE 5:** Effects of ZSS on the cardiovascular system.

Pharmacological effects	Extracts/Metabolites	Model	Dose range tested	Mechanism	Ref.
Cardiovascular effect	Jujuboside A	Mice	10, 20 mg/kg	--	Zhang et al. (2005)
	Spinosa	Wistar rats	0.1, 0.3 and 1.0 g/kg	Slowing down the heart rate to reduce the myocardium, oxygen consumption to reduce the damage of hypoxia to the myocardium; regulate the body‘s neuro-endocrine-immune system function to maintain internal environment stability; improve myocardial blood supply; recovery of myocardial contractility recovery	Zhang et al. (2005)
Lipid-regulating effect	Saponin	SD rats	120、240 and 480 mg/kg	The content of TC and TLDL-C in serum of hyperlipidemia animal model was decreased, the content of HDL-C was increased, and the ratio of TC/HDL was decreased	Chen et al. (1990)
Antihypertensive effect	Saponin	Rats	5, 10 and 25 mg/kg	--	Wu et al. (1991)

#### 3.2.1 Cardiovascular effects

Research data on the cardioprotective effects of ethanol extracts of ZSS indicated that intraperitoneal injection of ZSS solution (4 g/kg) and sublingual intravenous injection of ZSS solution (1.5 g/kg) both showed a tendency to improve myocardial ischemia induced by oxytocin in rats (Liu et al., 1985). Moreover, total saponins from ZSS (20 mg/kg) could significantly increase SOD activity in reperfused myocardial tissue, reduce MDA and LDH levels, and enhance the antioxidant capacity of myocardial cells, thereby improving myocardial ischemia (Huang et al., 2013). It was found that jujuboside A (20 mg/mL) could upregulate Bcl-2, downregulate Bax protein expression, increase Bcl-2/Bax ratio, inhibit the release of cytochrome C from mitochondria, reduce the activity of caspase-3 in myocardial tissue and reduce cardiomyocyte apoptosis (Huang, 2011).

#### 3.2.2 Lipid-lowering effect

Studies have shown that ZSS oil is rich in a variety of unsaturated fatty acids and has the effect of lowering blood lipids. Oral administration of ziziphus jujube oil (2.5 mL/kg) significantly reduced TC, LDL and TG, and increased High density lipoprotein/Low density lipoprotein (H/L) (Wu et al., 1991). In addition, it was also found that total saponins of ZSS (120 mg/kg) had similar effects on hyperlipidemia rats (Wu, 2004).

#### 3.2.3 Antihypertensive effect

The total saponins of ZSS (5 mg/kg) could show obvious antihypertensive effect on essential hypertensive rats at 0.5 h after administration. At present, the mechanism of its antihypertensive effect is not clear, which may be related to the synergistic effect of ZSS on myocardial hypoxia, myocardial ischemia and blood lipid regulation (Liu and Zhao, 2012).

### 3.3 Effect on the immune system

ZSS, as a commonly used traditional Chinese medicine, has various pharmacological effects such as immune regulation, anti-inflammation, and antioxidant properties. It plays a regulatory role in the immune system, enhancing the body’s immune function and improving its ability to resist diseases. The main components of ZSS include various active substances, among which polysaccharides are one of the important components responsible for its immune-regulating effects (Table 6).

**TABLE 6:** Effects of ZSS on the immune system.

Pharmacological effects	Extracts/Metabolites	Model	Dose range tested	Mechanism	Ref.
Anti-inflammatory effects	Jujuboside F	RAW 264.7	10 mM	Inhibits the release of pro-inflammatory cytokine TNF-α	Fu et al. (2017)
	Jujuboside G	RAW 264.7	10 mM	Inhibits the release of pro-inflammatory cytokine TNF-α	Fu et al. (2017)
	Jujuboside H	RAW 264.7	10 mM	Inhibits the release of pro-inflammatory cytokine TNF-α	Fu et al. (2017)
	Polysaccharides	Caco2 cells	200, 100, 50, 25 and 12.5 μg/mL	Regulating the assembly of tight junctions involves AMPK activation	Yue et al. (2015)
	Polysaccharides	RAW 264.7	200, 100, 50, 25 and 12.5 μg/mL	Regulating the assembly of tight junctions involves AMPK activation	Yue et al. (2015)
	Polysaccharides	Male SD rats	20, 40 and 80 mg/kg	Regulating the assembly of tight junctions involves AMPK activation	Yue et al. (2015)
Anti-tumor effect	Jujuboside B	AGS human gastric cancer cells	25, 50, 100 and 150 μM	Increase the activation of FasL and caspase-8. Activation of p38/c-Jun N-terminal kinase (JNK). Inhibition of exogenous pathway-mediated apoptosis by inducing protective autophagy	Xu et al. (2014)
	Jujuboside B	HCT 116 human colon cancer cells	25, 50, 100 and 150 μM	Increase the activation of FasL and caspase-8. Activation of p38/c-Jun N-terminal kinase (JNK). Inhibition of exogenous pathway-mediated apoptosis by inducing protective autophagy	Xu et al. (2014)
	Oil	Mice	1.4, 0.35 mL/kg	--	Wang et al. (1995)
Immunoregulation effect	Protein	ICR mice	100, 200 and 400 mg/kg	Activation of MAPKs and NF-κB signaling pathways	Zhang et al. (2023a)

#### 3.3.1 Anti-inflammatory effect

The effect of ZSS polysaccharide on colitis rats was studied. The results showed that ZSS polysaccharide (20 mg/kg) significantly improved the severity of colitis in colitis rats and inhibited the inflammatory response by reducing the activity of TNF-α, IL-1β, IL-6 and MPO in colitis rats (Yue et al., 2015). In addition, 10 dammarane saponins isolated from ZSS could significantly inhibit the activity of pro-inflammatory cytokine TNF-α in LPS-induced RAW 264.7 macrophages. At 50 μM, the inhibition rate of jujuboside I was up to 42.6% (Fu et al., 2015).

#### 3.3.2 Antitumor effect

By studying the effect of ZSS oil on the survival time and body weight of Ehrlich ascites carcinoma mice, it was proved that ZSS oil (0.35 mL/kg) could significantly prolong the survival days of tumor-bearing mice, the life extension rate was more than 50%, and the weight gain of tumor-bearing mice in the later stage of life was significantly inhibited, indicated that ZSS oil had a significant tumor-bearing effect on Ehrlich ascites carcinoma mice (Wang et al., 1995). Another study showed that jujuboside B (40 mg/kg) could induce apoptosis and autophagy of AGS and HCT 116 human cancer cells, and effectively inhibit the growth of subcutaneous xenografts of HCT 116 cells in nude mice. Mechanism studies have shown that jujuboside B induced apoptosis is related to exogenous pathways by increasing the activation of Fasl and caspase-8 (Xu et al., 2014).

#### 3.3.3 Immunoregulation effects

A study showed that the alcohol extract of ZSS (5 g/kg) could increase the appetite of mice, increase the body weight of mice, significantly enhance the delayed type hypersensitivity (DH) of mice, and antagonize the inhibition of DH induced by CPA in mice, showed an immune enhancement effect (Lang et al., 1988). In the study of the immune enhancement effect of polysaccharide of ZSS, it was found that polysaccharide (0.1 g/kg) could significantly promote the proliferation of RAW 264.7 cells, promote the release of NO and the expression of stress response proteins (COX-2 and iNOS) in RAW 264.7 cells. In addition, polysaccharide could also significantly promote the phosphorylation of IkB-α and ERK proteins (Lang et al., 1991).

### 3.4 Other pharmacological effects

In addition to the above pharmacological effects, ZSS also has other pharmacological effects (Table 7). 6‴-p-coumaroylspinosin (P-CS) is a flavonoid component from ZSS. It was found that P-CS (20 nM) prevented acrylamide (AA) induced cell death, reduced glutathione (GSH) content and excessive production of reactive oxygen species (ROS) by inhibiting AA induced Bax and Bim expression and inhibiting the JNKs pathway, confirmed its significant antioxidant activity (Li et al., 2020). A study found that jujuboside A (Ju A) (5 mg/kg) can alleviate type 2 diabetes (T2DM) related liver injury by reducing liver lipid accumulation, inflammatory response and oxidative stress in the liver of db/db mice (Zhang et al., 2024). In another study, jujuboside B (JuB) was found to have similar pharmacological effects. JuB (20 mg/kg) could reverse the accumulation of CYP2E1 induced by APAP, inhibit oxidative stress and proinflammatory cytokines in mice with liver injury, and reduce hepatocyte apoptosis. Its function was related to the regulation of Nrf2-STING pathway (Wang et al., 2024).

**TABLE 7:** Other pharmacological effects of ZSS.

Pharmacological effects	Extracts/Metabolites	Model	Dose range tested	Mechanism	Ref.
Antioxidation effect	6‴-p-Coumaroylspinosin	PC12 cells	20, 50, 100, 200, 300, and 400 nM	Inhibition of acrylamide induced Bax and Bim expression and JNKs pathway	Li et al. (2020)
	6‴-O-Acetylsespinorolactone	*Caenorhabditis elegans*	10, 100 and 200 µM	Reduce the level of ROS and MDA, enhance the activity of GSH-Px, increase the expression of SOD-3 and GST-4, and improve the ability of antioxidant damage	Zhang et al. (2023b)
Hepatoprotective effect	Jujuboside A	Mice	5, 10 and 20 mg/kg	Inhibition of YY1/CYP2E1 signaling alleviates T2DM-related NAFLD by activating PPARα	Zhang et al. (2024)
	6‴-O-Acetylsespinorolactone	HepG2 cells	1.25, 2.5 and 5 μM	Inhibition of YY1/CYP2E1 signaling alleviates T2DM-related NAFLD by activating PPARα	Wang et al. (2024)

## 4 Pharmacokinetics

The study of pharmacokinetics is of great significance. It can not only reveal the mechanism of action of drugs and optimize drug treatment regimens, but also predict the efficacy and toxicity of drugs, achieve individualized drug treatment, and improve the efficiency of new drug research and development. At present, there are few studies on the pharmacokinetics of ZSS extract.

An ultra-performance liquid chromatography-tandem mass spectrometry (UPLC-MS/MS) method was developed for the simultaneous determination of the pharmacokinetics of nine chemical components in beagle dog plasma following the administration of fried ZSS extract. After a single dose of fried ZSS extract, the T_max_ and t_1/2_ of magnoflorine, aconitine, vicenin II, isospirin, spinosin, 6‴-feruloylspinosin, and jujuboside B were 2.40–3.20 h and 2.08–6.79 h, respectively. The exposure of magnoflorine and spinosin were relatively high, with Cmax of 76.7 and 31.5 ng/mL, AUC_0-∞_ of 581 and 315 ng h/mL, respectively. The C_max_ of the other five compounds, which exhibited lower exposure, ranged from 0.81 to 13.0 ng/mL, with AUC_0-∞_ values between 6.00 and 106 ng h/mL. A double peak phenomenon may occur in beagle dogs for spinosin, isospirin, and 6‴-feruloylspinosin. However, this study did not further investigate whether the double-peak absorption of these compounds is due to hepatointestinal circulation, dual-site absorption, or the hydrolysis of glycosidic bonds by intestinal hydrolases, leading to the reabsorption of the resulting aglycones by the intestine (Xia et al., 2024).

The pharmacokinetics and tissue distribution of Spinosin in rats after intravenous administration were investigated by HPLC. The time-concentration curve after a single intravenous injection of 20 mg/kg Spinosin in rats conformed to the two-compartment model. The main pharmacokinetic parameters, including the half-lives (T_0.5α_ and T_0.5β_), clearance (CLs), area under the curve (AUC_0-T_), and volume of distribution (Vd), were 6.66 min, 51.5 min, 1.42 L/min, 2.83 mg min/mL, and 14.0 L/kg, respectively. At 20 min post-administration, the peak concentration was observed in the liver and brain tissues. Spinosin exhibited the highest concentration in the liver, followed by the spleen and kidneys. Spinosin exhibited the highest concentration in the liver, followed by the spleen and kidneys. The lowest concentration of Spinosin was found in the testes, followed by the brain, while it was not detected in smooth or skeletal muscle. After intravenous administration, the drug was widely distributed in the rats and transported rapidly (Li et al., 2007).

It has been reported in the literature that Spinosin exhibits bimodal absorption in both healthy rats and rats with insomnia models. From the perspective of intestinal flora metabolism and pharmacokinetics, Duan et al. elucidated the processes of absorption, metabolism, and transformation of Spinosin and its derivatives in rats. The pharmacokinetic characteristics of Spinosin in rat plasma were thoroughly investigated. The prototype components and metabolites in feces were analyzed using UHPLC-Q-TOF-MS/MS. The concentration of Spinosin in rat plasma was determined by UPLC-MS/MS. The pharmacokinetics of Spinosin in rats were examined, revealing that Spinosin derivatives, such as 6″-p-coumaroylspinosin and 6″-feruloylspinosin, could be converted into Spinosin by β-glucosidase produced by rat intestinal flora *in vitro*. Additionally, the intestinal flora can effectively hydrolyze the p-coumaroyl, feruloyl, and p-hydroxybenzoyl groups of Spinosin derivatives to form a common metabolite, Spinosin, which can further be hydrolyzed to produce swertisin (6-C sugar) (Duan et al., 2022). This may be the primary reason for the bimodal absorption of Spinosin.

## 5 Toxic side effects

Due to its effective sedative hypnotic properties, the pharmacological activity of ZSS has garnered increasing attention from researchers. This substance is among the first batch of dual-use medicinal and edible products approved by the Ministry of Health of China. To date, few toxic reactions have been reported in dietary or clinical applications, and it is regarded as a highly safe traditional Chinese medicine. An oral toxicity test of Ziziphus jujuba root extract revealed no toxicity or mortality at a dosage of 2,500 mg/kg, indicated its safety for consumption (Alam et al., 2016).

A study investigating the toxicological characteristics of Tianma and Suanzaoren compound capsules revealed that after intragastric administration of the maximum dose (30,000 mg/kg), no signs of poisoning or mortality were observed in mice. The results of the Ames test, mouse bone marrow cell micronucleus test, and mouse sperm deformity test were all negative. The 30-day feeding trial indicated no significant differences in the parameters of rats across all dose groups compared to the control group (*p* > 0.05). These findings suggested that the Tianma Suanzaoren capsule was non-toxic and did not exhibit genotoxicity within the parameters of this experiment (Yuan et al., 2013).

In addition, during the acute and sub-chronic toxicity tests of ZSS oral liquid, the acute toxicity test did not determine the median lethal dose of the ZSS oral liquid, and the maximum tolerance observed in chicks exceeded 20 g/kg. In the sub-chronic toxicity test, the body weight of the high-dose group (5 g/kg) was significantly different from that of the control group on the seventh day (*p* < 0.01); however, this difference was not significant on the 28th day. This finding indicated that the effects of ZSS oral liquid on blood biochemical indices were reversible, suggested that continuous oral administration is safe (Li et al., 2010).

The objective of this study was to evaluate the acute toxicity of the alcohol extract of ZSS. It was observed that some mice exhibited signs of poisoning and died following intravenous injection of the ethanol extract. The calculated LD50 was 27.5 g/kg, with a 95% confidence interval ranging from 25.1 to 30.1 g/kg. No pathological changes were detected in the major organs of the mice. Additionally, after intragastric administration at a dosage of 340 g/kg, all mice survived after 14 days of continuous observation, and no significant toxic reactions were noted. These findings indicated that the toxicity of the ethanol extract of ZSS was very low, suggested that its clinical administration was both safe and reliable (Wang L. J. et al., 2009). At present, there are few studies on the toxicity of ZSS extract and its chemical components, but the comprehensive experimental results show that the toxicity of ZSS is very low and the clinical medication is safe.

## 6 Conclusion and future perspectives

ZSS is primarily distributed across various provinces and regions in northwest, northeast, and northern China, as well as in certain areas of southern China. Due to its notable effects on sleep regulation, it has earned the nickname sleeping fruit. ZSS is rich in flavonoids, saponins, alkaloids, organic acids, and other beneficial metabolites. It is known for its ability to nourish the heart and liver, calm the mind, and promote tranquility. This substance is among the first batch of and food homologous substances recognized in China (Zhang et al., 2022). Modern research has demonstrated that the active ingredients in ZSS can regulate the nervous, cardiovascular, hematopoietic, and immune systems, as well as material metabolism. Additionally, these compounds exhibit pharmacological effects such as antioxidant, anti-aging, and anti-cancer properties (Wang et al., 2022). However, a review of the literature reveals that there are still several issues in the research surrounding ZSS.

Although research on the pharmacological effects of ZSS has been relatively comprehensive, recent studies have rarely delved deeply into its anti-inflammatory, anti-lipid peroxidation, and antihypertensive properties. Furthermore, investigations into the active components of ZSS primarily focus on saponins, flavonoids, and alkaloids, while the effects of fatty oils and other chemical constituents remain underreported (Wan et al., 2008). It has been reported that ZSS is rich in protein, with a protein content of approximately 27%. Protein serves as a primary source of nutrition for both humans and animals. As health awareness increases, people are becoming more cognizant of the potential side effects associated with excessive consumption of animal-derived protein, such as hyperlipidemia and obesity. Consequently, plant-derived protein has emerged as a recognized source of which are easily absorbed by the human body and exhibit strong functionality. This topic has gained significant attention in the field of food research (Rodsamran and Sothornvit, 2018; Zhang et al., 2020). Therefore, it is of great significance to develop and utilize the resources of ZSS protein, explore its potential medicinal value, and systematically investigate the biological activity of this protein. Additionally, there are limited reports on the pharmacokinetics and toxicology of ZSS. Consequently, studying ZSS can enhance the in-depth exploration of these aspects and provide a foundation for the safety and reliability of its clinical use.
