# Supplementary material for: Phytochemical, pharmacological, pharmacokinetic and toxicological characteristics of Ziziphi Spinosae Semen: a review
Source: Front Pharmacol. 2024 Nov 29;15:1504009. doi: 10.3389/fphar.2024.1504009 (PMC11639084; doi:10.3389/fphar.2024.1504009)
Supplement: Supplementary file 1 [file DataSheet1.zip › Supplementary_Material/Supplementary_Material.docx]

Supplementary Material

# Supplementary Figures and Tables

## Supplementary Figures


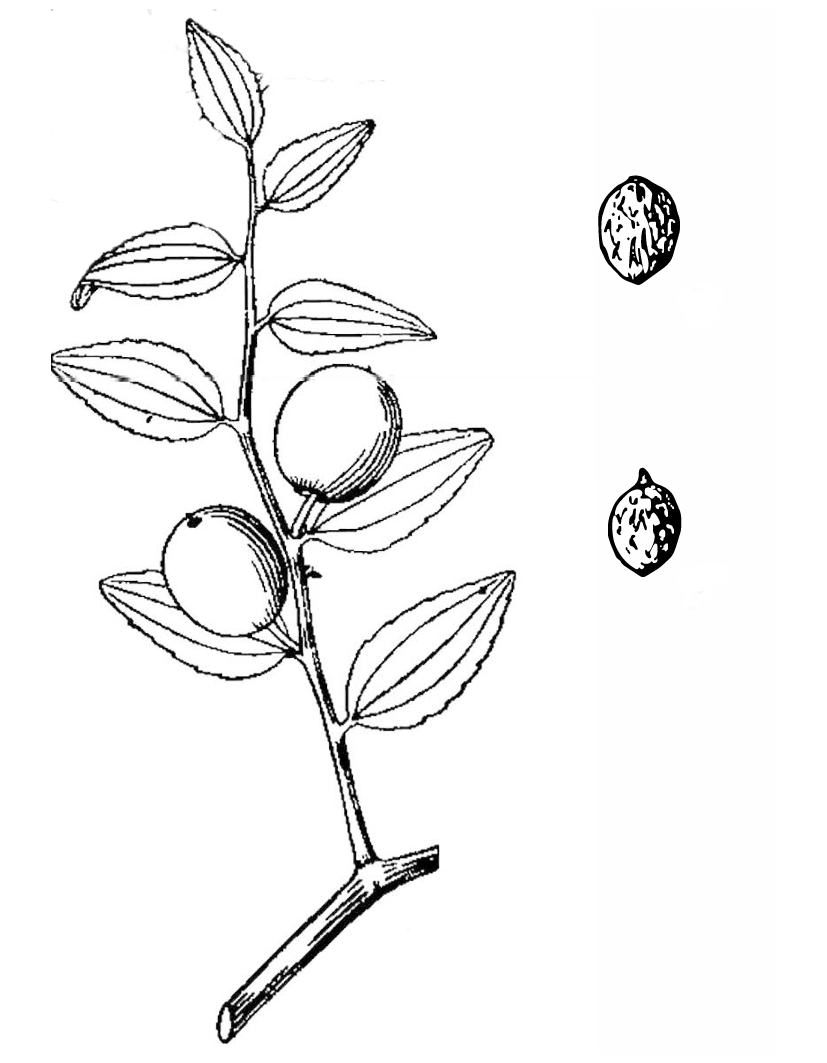


**Supplementary Figure 1.** Plant diagram of *Ziziphi Spinosae Semen*.


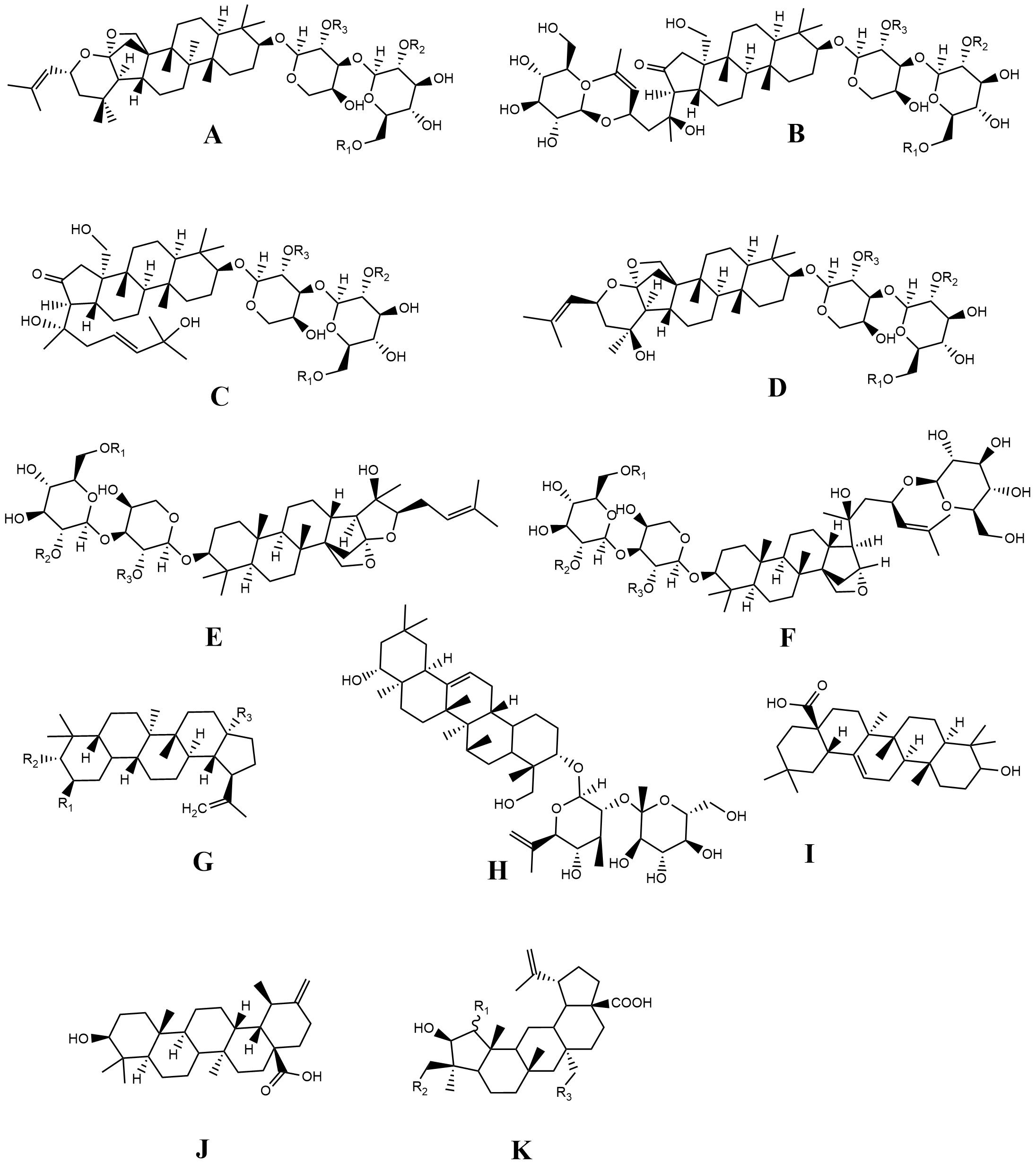


**Supplementary Figure 2.** Chemical structures of the saponins in ZSS (1-33).


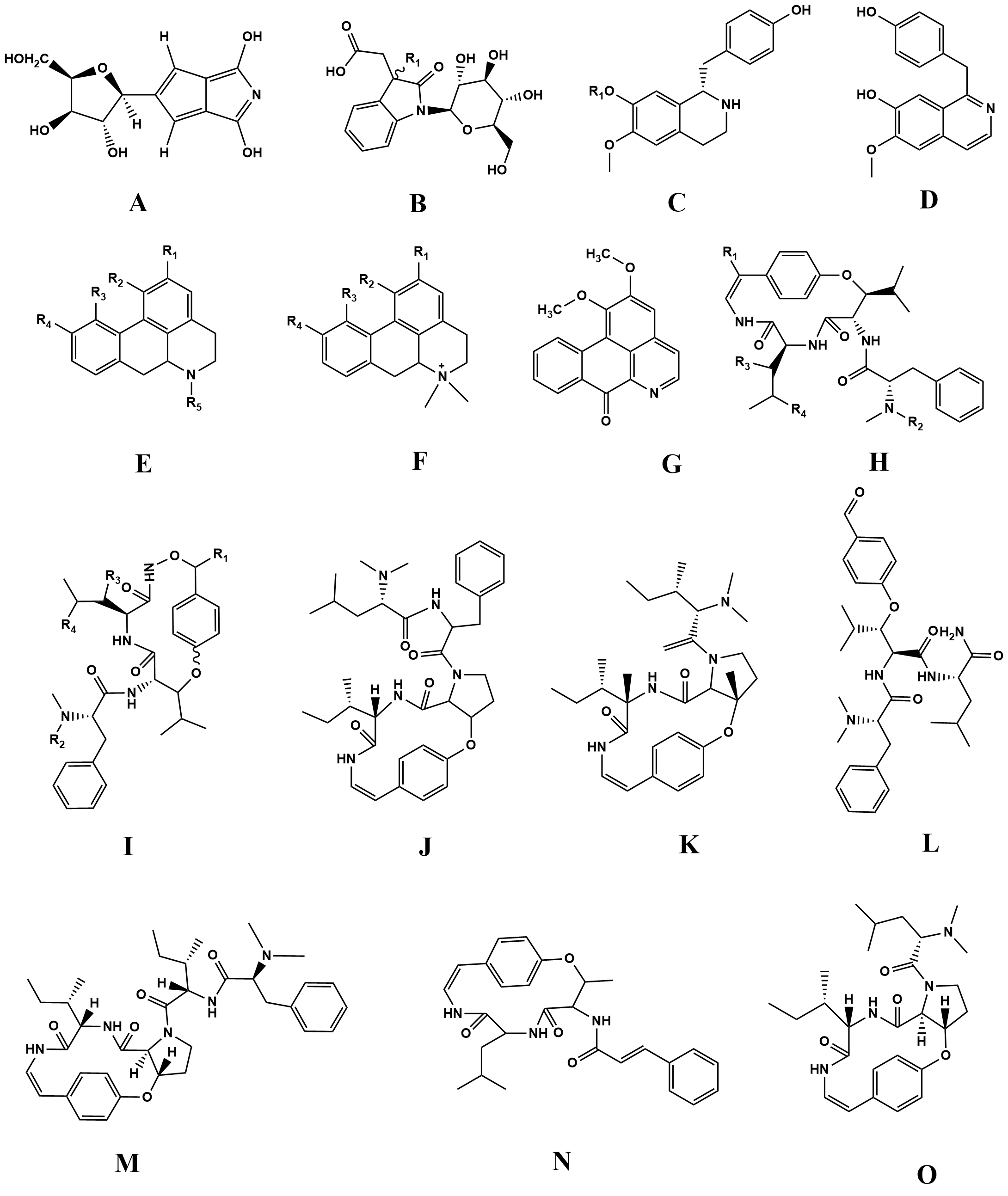


**Supplementary Figure 3.** Chemical structures of the alkaloids in ZSS (34-59).


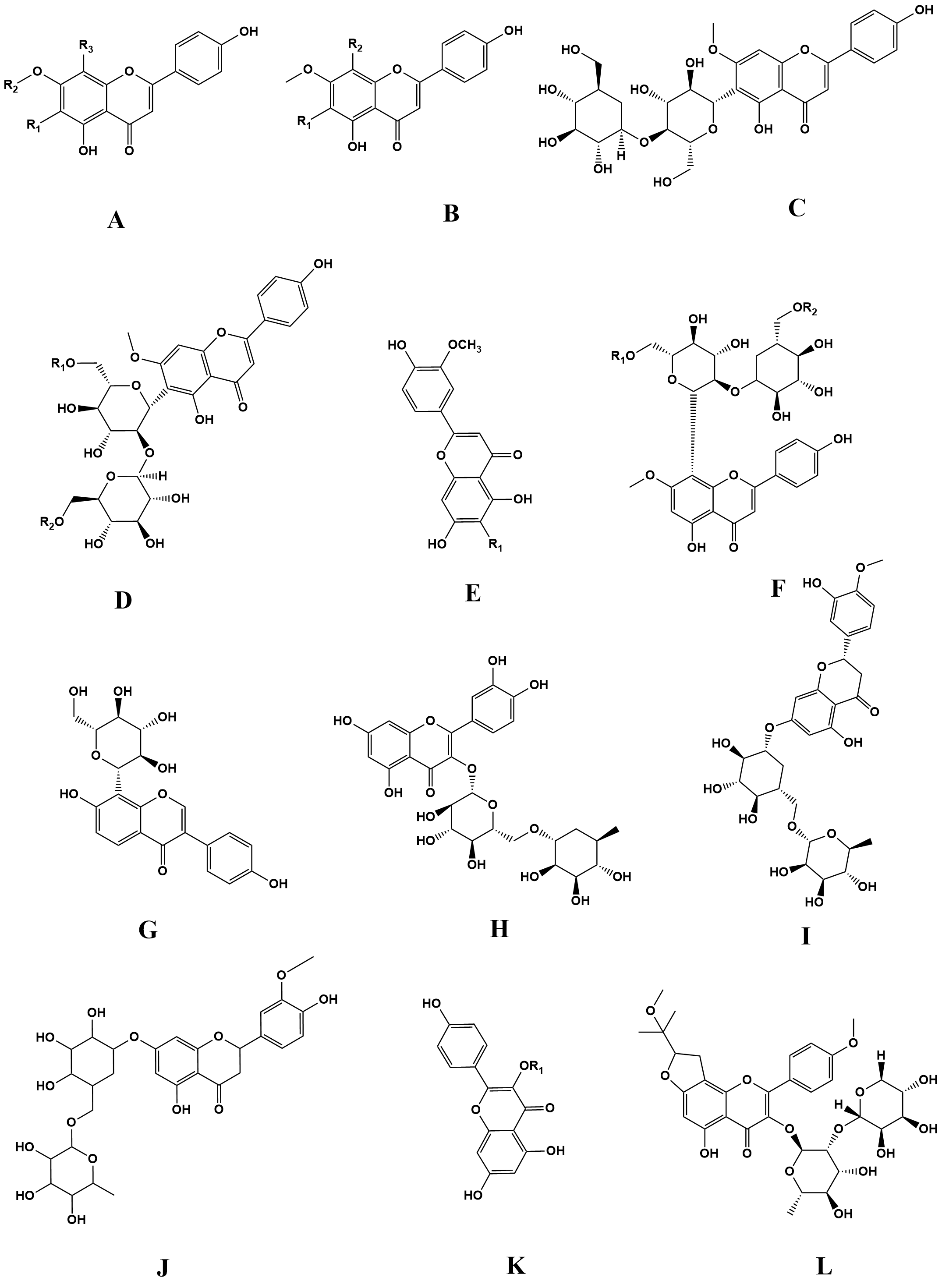


**
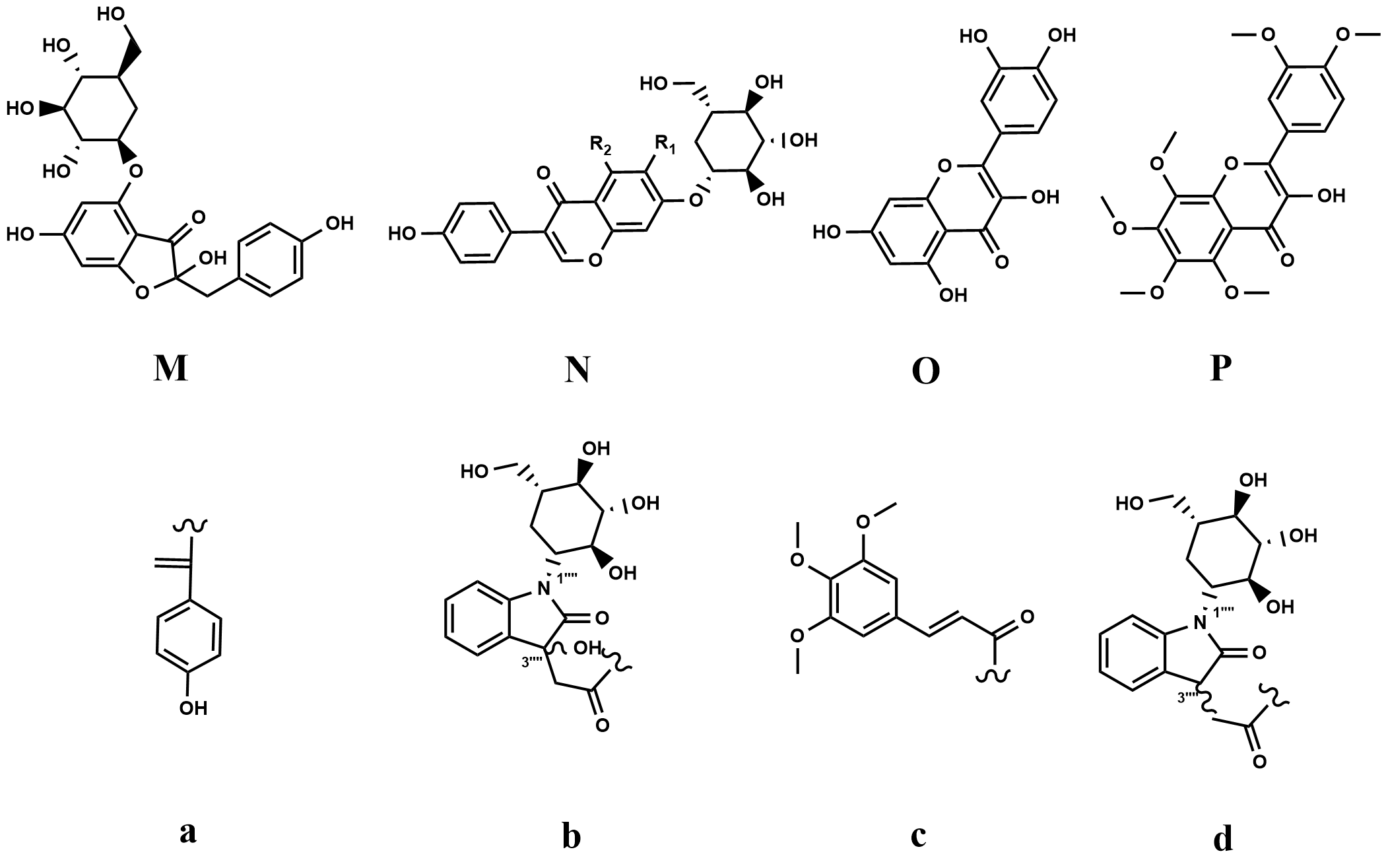
**

**Supplementary Figure 4.** Chemical structures of the flavonoids in ZSS (60-109).


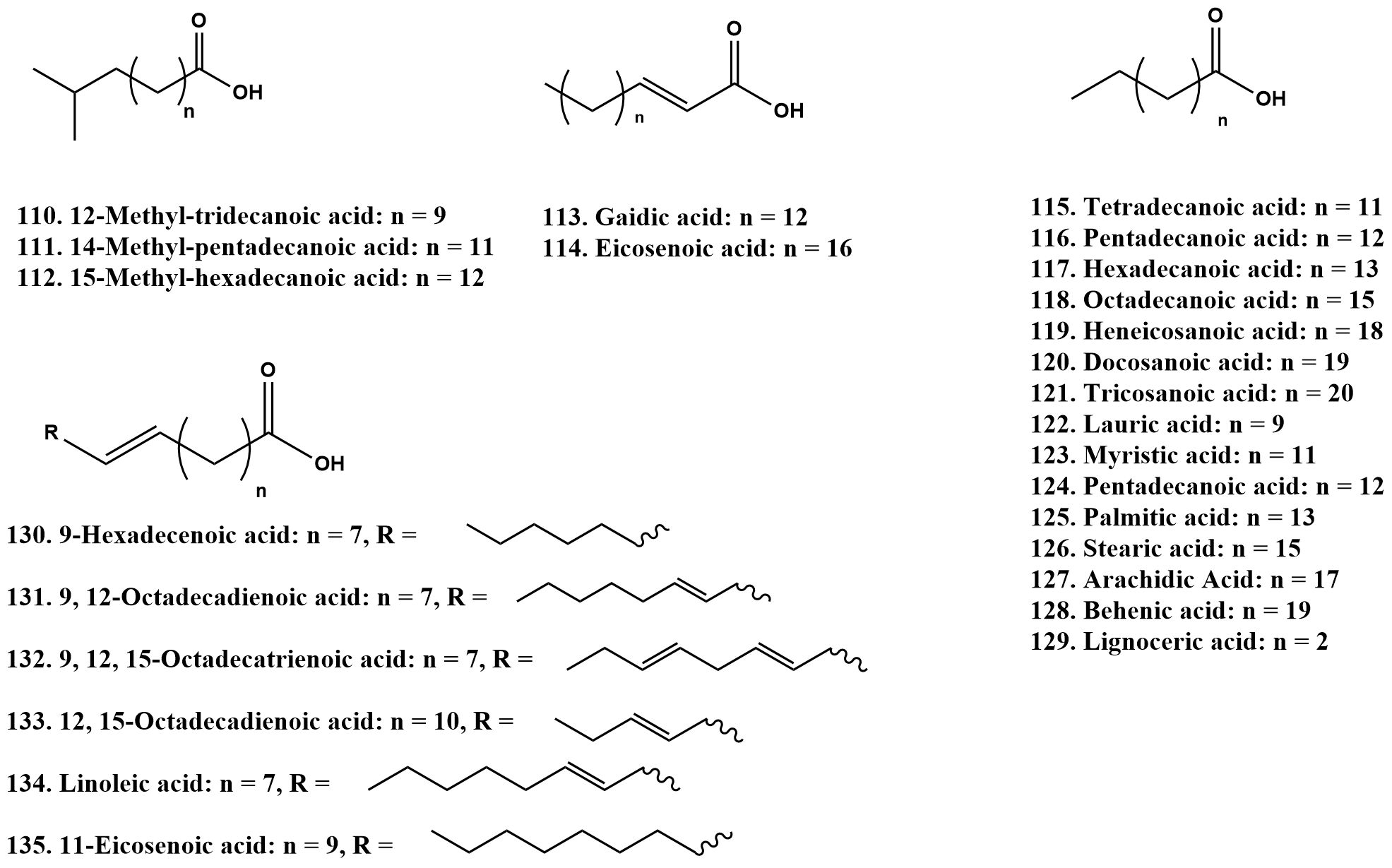


**Supplementary Figure 5.** Chemical structures of the fatty acids and volatile oils in ZSS (110-135).


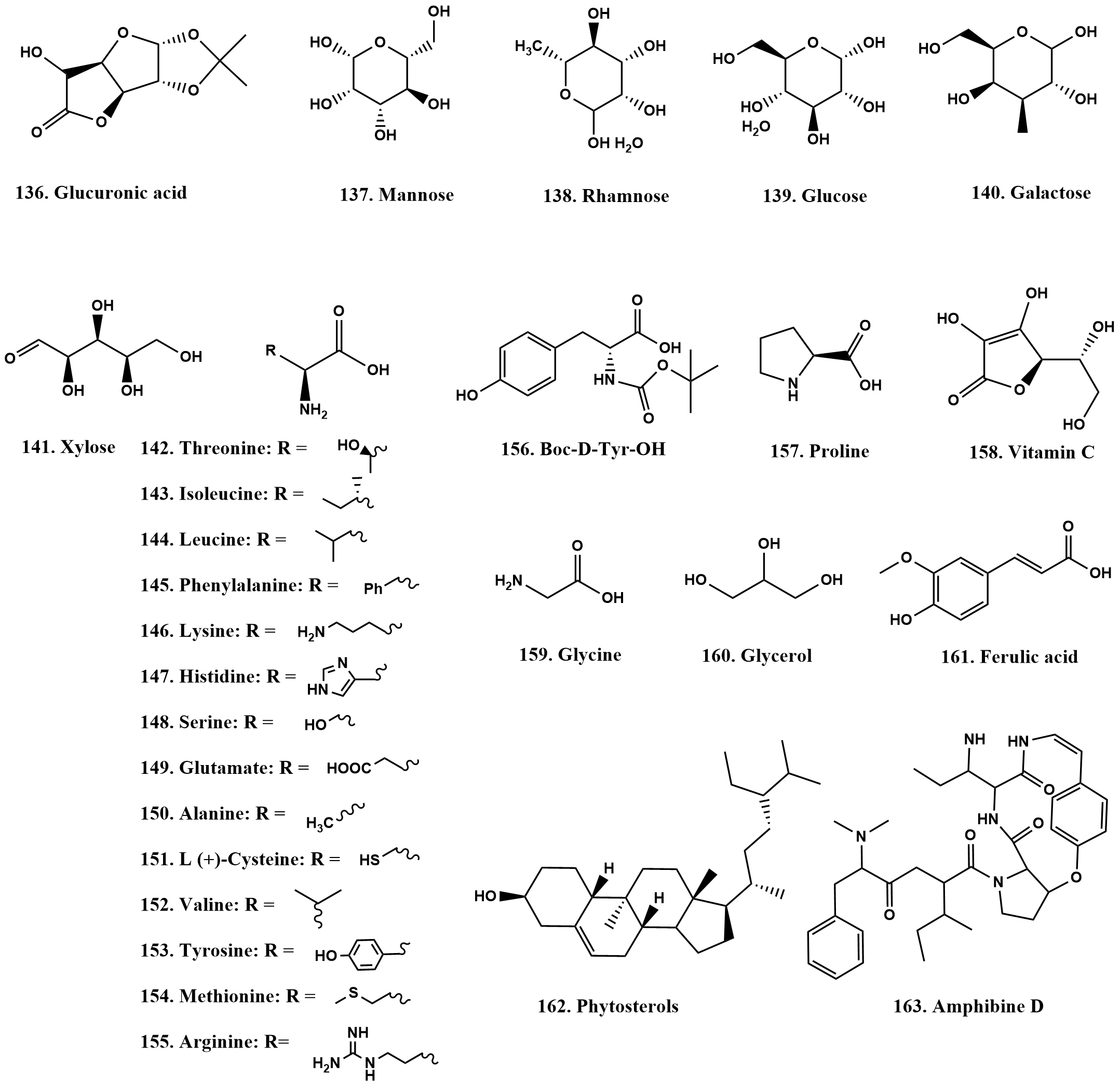


**Supplementary Figure 6.** Chemical structures of other compounds in ZSS (136-163).

## Supplementary Tables

**Supplementary Table 1.** Saponins isolated from ZSS.

| NO. | Metabolites | Core | substituent | | | Ref. |
| --- | --- | --- | --- | --- | --- | --- |
|  |  |  | R1 | R2 | R3 |  |
| 1 | Jujuboside A | A | glc | xyl | rha | (Otsuka et al., 1978) |
| 2 | Jujuboside A1 | A | glc | xyl | fuc | (Matsuda et al., 1999; Liu et al., 2004) |
| 3 | Jujuboside A2 | A | glc | xyl | 6-deoxy-talose | (Liu et al., 2004) |
| 4 | Jujuboside B | A | H | xyl | rha | (Otsuka et al., 1978) |
| 5 | Jujuboside B1 | A | H | xyl | fuc | (Matsuda et al., 1999) |
| 6 | Jujuboside C | A | glc | glc | rha | (Matsuda et al., 1999) |
| 7 | Acetyljujuboside B | A | Ac | xyl | rha | (Matsuda et al., 1999) |
| 8 | Zizyphus saponin II | A | H | H | rha | (Wang et al., 2013) |
| 9 | Zizyphus saponin III | A | H | xyl | 6-deoxy-talose | (Liu. 2013) |
| 10 | Jujuboside I | A | H | glc | rha | (Wang et al., 2013) |
| 11 | Protojujuboside A | B | glc | xyl | rha | (Matsuda et al., 1999) |
| 12 | Protojujuboside B | B | H | xyl | rha | (Matsuda et al., 1999) |
| 13 | Protojujuboside B1 | B | H | xyl | fuc | (Matsuda et al., 1999) |
| 14 | Jujuboside H | C | glc | xyl | rha | (Wang et al., 2009a) |
| 15 | Jujuboside G | C | H | xyl | rha | (Wang and Yang. 2008) |
| 16 | Jujuboside II | D | H | xyl | rha | (Wang et al., 2013) |
| 17 | Jujuboside III | E | H | xyl | rha | (Wang et al., 2013) |
| 18 | Jujuboside IV | E | glc | xyl | rha | (Wang et al., 2013) |
| 19 | Jujuboside E | F | glc | xyl | rha | (Bai et al., 2003) |
| 20 | Betulonic acid | G | H | OH | COOH | (Zhang et al., 2016) |
| 21 | Alphitolic aicd methyl ester | G | OH | OH | COOCH_3_ | (He et al., 2006) |
| 22 | Lupeol | G | H | OH | CH_3_ | (Wang and Yang. 2008) |
| 23 | Methyl betulinate | G | H | OH | COOCH_3_ | (Wang and Yang. 2008) |
| 24 | Betulin | G | H | OH | CH_2_OH | (Zeng et al., 1986) |
| 25 | Alphitolic acid | G | OH | OH | COOH | (Zhang et al., 2016) |
| 26 | Betulinic acid | G | H | OH | COOH | (Zeng et al., 1986) |
| 27 | Azukisaponin Ⅱ | H | -- | -- | -- | (Li et al., 2005) |
| 28 | Oleanolic acid | I | -- | -- | -- | (Cao and Wang, 2009) |
| 29 | Zizyphursolic acid | J | -- | -- | -- | (Yang et al., 2013) |
| 30 | 24-Hydroxyceanothic acid | K | αCOOH | OH | H | (Zhang et al., 2016) |
| 31 | 27-Hydroxyceanothic acid | K | αCOOH | H | OH | (Zhang et al., 2016) |
| 32 | Ceanothic acid | K | αCOOH | H | H | (Zeng et al., 1986) |
| 33 | Epiceanothic acid | K | βCOOH | H | H | (Zhang et al., 2016) |

**Supplementary Table 2.** Alkaloid isolated from ZSS.

| NO. | Metabolites | Core | substituent | | | | | Ref. |
| --- | --- | --- | --- | --- | --- | --- | --- | --- |
|  |  |  | R1 | R2 | R3 | R4 | R5 |  |
| 34 | 6-(2′,3′-dihydroxy-4′-hydroxymethyl-tetrahydro-furan-1′-yl)-Cyclopentene [C] pyrrole-1,3-diol | A | -- | -- | -- | -- | -- | (Xie et al., 2011) |
| 35 | 3*S*-1-*N*-β-*D*-Glucopyranosyl-2-oxo-3-hydroxy-indole-3-acetic acid | B | OH(S) | -- | -- | -- | -- | (Li et al., 2014) |
| 36 | 3*R*-1-*N*-β-*D*-Glucopyranosyl-2-oxo-3-hydroxy-indole-3-acetic acid | B | OH(R) | -- | -- | -- | -- | (Xie et al., 2011) |
| 37 | Coclaurine | C | -- | -- | -- | -- | -- | (Mesmar et al., 2022) |
| 38 | Juzirine | D | -- | -- | -- | -- | -- | (Yin et al., 1997) |
| 39 | Norisocorydine | E | OCH_3_ | OCH_3_ | OH | OCH_3_ | H | (Han et al., 1989) |
| 40 | Caaverine | E | OCH_3_ | OH | H | H | H | (Mesmar et al., 2022) |
| 41 | Asimilobine | E | OH | OCH_3_ | H | H | H | (Yin et al., 1997) |
| 42 | Nornuciferine | E | OCH_3_ | OCH_3_ | H | H | H | (Mesmar et al., 2022) |
| 43 | *N*-Methylasimilobine | E | OH | OCH_3_ | H | H | CH_3_ | (Han et al., 1989) |
| 44 | Nuciferine | E | OCH_3_ | OCH_3_ | H | H | CH_3_ | (Han et al., 1989) |
| 45 | Zizyphusine | F | OH | OH | OCH_3_ | OCH_3_ | -- | (Mesmar et al., 2022) |
| 46 | Magnoflorine | F | OCH_3_ | OH | OH | OCH_3_ | -- | (Lee et al., 2012) |
| 47 | Lysicamine | G | -- | -- | -- | -- | -- | (Yin et al., 1997) |
| 48 | 6-Glc-coclaurine | C | Glu | -- | -- | -- | -- | (Zhang et al., 2016) |
| 49 | Sanjoinine A | H | H | CH_3_ | H | CH_3_ | -- | (Mesmar et al., 2022) |
| 50 | Sanjoinine F | H | H | CH_3_ | OH | CH_3_ | -- | (Mesmar et al., 2022) |
| 51 | Sanjoinine B | H | H | H | H | CH_3_ | -- | (Mesmar et al., 2022) |
| 52 | Sanjoinine D | I | H | CH_3_ | CH_3_ | H | -- | (Mesmar et al., 2022) |
| 53 | Sanjoinine G1 | I | OCH_3_ | CH_3_ | H | CH_3_ | -- | (Xie et al., 2011) |
| 54 | Lotusine B | J | -- | -- | -- | -- | -- | (Yin et al., 1997) |
| 55 | Ramosine A | K | -- | -- | -- | -- | -- | (Yin et al., 1997) |
| 56 | Anjoinine G2 | L | -- | -- | -- | -- | -- | (Mesmar et al., 2022) |
| 57 | Amphibine D | M | -- | -- | -- | -- | -- | (Han et al., 1990) |
| 58 | Sanjoinenine | N | -- | -- | -- | -- | -- | (Han et al., 1989) |
| 59 | Mucronine J | O | -- | -- | -- | -- | -- | (Mesmar et al., 2022) |

**Supplementary Table 3.** Alkaloid isolated from ZSS.

| NO. | Metabolites | Core | substituent |  |  | Ref. |
| --- | --- | --- | --- | --- | --- | --- |
|  |  |  | R1 | R2 | R3 |  |
| 60 | Apigenin-6-C-glucopyranoside | A | Glc | H | H | (Cheng et al., 2000) |
| 61 | Isovitexin-2″-O-β-D-glucopyranoside | A | Glc-glc | H | H | (Cheng et al., 2000) |
| 62 | Isovitexin-2″-O-(6-p-coumaloyl)-glucopyranoside | A | Glc-glc-*p*-coumaroyl | H | H | (Zhang et al., 2016) |
| 63 | Isovitexin-2″-O-(6-feruloyl)-glucopyranoside | A | Glc-glc-feruloyl | H | H | (Zhang et al., 2016) |
| 64 | Vitexin | A | H | H | Glc | (Cheng et al., 2000) |
| 65 | Vicenin II | A | gGlc | H | Glc | (Zhang et al., 2016) |
| 66 | Swertisin | B | Glc | H | -- | (Xie et al., 2011) |
| 67 | Zivulgarin | C | -- | -- | -- | (Wu et al., 2011) |
| 68 | Spinosin | D | H | H | -- | (Woo et al., 1979) |
| 69 | 6′′′-Vanilloylspinosin | D | H | vanilloy | -- | (Wu et al., 2011) |
| 70 | 6′′′-(4′′′′-O-β-D-glucopyranosyl)-Vanilloylspinosin | D | H | Vanilloy-glc | -- | (Xie et al., 2011) |
| 71 | 6′′′-Dihydrophaseoylspinosin | D | H | dihydrophaseoyl | -- | (Zhang et al., 2012) |
| 72 | 6′′′-Sinapoylspinosin | D | H | sinapoyl | -- | (Woo et al., 1980) |
| 73 | 6′′′-p-Coumaloylspinosin | D | H | P-coumaroyl | -- | (Woo et al., 1980) |
| 74 | 6′′′-Feruloylspinosin | D | H | feruloyl | -- | (Woo et al., 1980) |
| 75 | 6″,6′′′-Diferuloylspinosin | D | feruloyl | feruloyl | -- | (Fu et al., 2017) |
| 76 | 6″-O-Feruloylspinosin | D | feruloyl | H | -- | (Fu et al., 2017) |
| 77 | 6″-O-Feruloyl-6′′′-p-hydroxybenzoylspinosin | D | feruloyl | a | -- | (Fu et al., 2017) |
| 78 | 6′′′-O-(3S-1-N-β-D-glucopyranosyl-2-oxo-3-hydroxy-indole-3-acetyl)  Spinosin | D | H | b(3””-S) | -- | (Li et al., 2014) |
| 79 | 6′′′-O-(3R-1-N-β-D-glucopyranosyl-2-oxo-3-hydroxy-indole-3-acetyl) Spinosin | D | H | b(3””-R) | -- | (Li et al., 2014) |
| 80 | 6″-O-(3S-1-N-β-D-glucopyranosyl-2-oxo-3-hydroxy-indole-3-acetyl) Spinosin | D | b(3””-S) | H | -- | (Li et al., 2014) |
| 81 | 6″-O-(3R-1-N-β-D-glucopyranosyl-2-oxo-3-hydroxy-indole-3-acetyl)  Spinosin | D | b(3””-R) | H | -- | (Li et al., 2014) |
| 82 | 6″-O-(3S-1-N-β-D-glucopyranosyl-2-oxo-3-hydroxy-indole-3-acetyl)-6′′′-  Feruloylspinosin | D | b(3””-S) | feruloyl | -- | (Li et al., 2014) |
| 83 | 6″-O-(3R-1-N-β-D-glucopyranosyl-2-oxo-3-hydroxy-indole-3-acetyl)-6′′′-  Feruloylspinosin | D | b(3””-R) | feruloyl | -- | (Li et al., 2014) |
| 84 | 6′′′-p-Hydroxybenzoylspinosin | D | H | P-hydroxybenzoyl | -- | (Wu et al., 2015) |
| 85 | 6′′′-(3′′′′,4′′′′,5′′′′-trimethoxyl)-(E)-Cinnamoylspinosin | D | H | c | -- | (Zhang et al., 2016) |
| 86 | 6′′′-(–)-Phaseoylspinosin | D | H | phaseoyl | -- | (Zhang et al., 2016) |
| 87 | 6′′′-(4′′′′-O-β-D-glucopyranosyl)-Benzoylspinosin | D | H | 4-O-glc-benzoyl | -- | (Zhang et al., 2016) |
| 88 | 6″-Feruloyl-6′′′-vanillyolspinosin | D | feruloyl | vanilloy | -- | (Chen et al., 2015) |
| 89 | 6′′′-(N-β-D-glucopyranosyl)-2′′′′,3′′′′-Dihydro-2′′′′-oxo-3′′′′-yl-acetate spinosin | D | H | d(3””-S) | -- | (Wu et al., 2015) |
| 90 | Epi-6′′′-(N-β-D-glucopyranosyl)-2′′′′,3′′′′-dihydro-2′′′′-oxo-3′′′′-yl-acetate  spinosin | D | H | d(3””-R) | -- | (Wu et al., 2015) |
| 91 | Isoscoparin-2″-O-(6-feruloyl)-glucopyranoside | E | Glc-glc-feruloyl | -- | -- | (Zhang et al., 2016) |
| 92 | Isospinosin | F | H | H | -- | (Cheng et al., 2000) |
| 93 | 6′′′-Feruloylisospinosin | F | H | feruloyl | -- | (Cheng et al., 2000) |
| 94 | 6″,6′′′-Diferuloylisospinosin | F | feruloyl | feruloyl | -- | (Zhang et al., 2016) |
| 95 | Isowertisin | B | H | Glc | -- | (Wang et al., 2008) |
| 96 | Puerarin | G | -- | -- | -- | (Cheng et al., 2000) |
| 97 | Naringin | A | H | Glc-rha | H | (Zhang et al., 2015) |
| 98 | Rutin | H | -- | -- | -- | (Zhang et al., 2015) |
| 99 | Hesperidin | I | -- | -- | -- | (Xie et al., 2011) |
| 100 | Clematine | J | -- | -- | -- | (Xie et al., 2011) |
| 101 | Nicotiflorin | K | Glc-rha | -- | -- | (Zhang et al., 2016) |
| 102 | Camelliaside B | K | Glc-rha (6”)-xyl(2”) | -- | -- | (Li et al., 2005) |
| 103 | Spinorhamnoside | L | -- | -- | -- | (Lee et al., 2012) |
| 104 | Hovetrichoside C | M | -- | -- | -- | (Li et al., 2005) |
| 105 | Glycitin | N | OCH_3_ | H | -- | (Wang et al., 2005) |
| 106 | Genistin | N | H | OH | -- | (Wang et al., 2005) |
| 107 | Quercetin | O | -- | -- | -- | (Cao and Wang, 2009) |
| 108 | 5,6,7,8,3′,4′-Hexamethoxy | P | -- | -- | -- | (Wang et al., 2005) |
| 109 | Apigenin | A | H | H | H | (Zhang et al., 2015) |

**Supplementary Table 4.** Effects of ZSS on the nervous system.

| Pharmacological  effects | Extracts/Metabolites | Model | Dose range tested | Mechanism | Ref. |
| --- | --- | --- | --- | --- | --- |
| Sedative hypnotic effect | SZJ-I and SZJ-II | Male ICR mice | 20, 40 and 80 mg/kg | The expression of 5-hydroxytryptamine 1A (5-HT1A), 5-hydroxytryptamine 2A (5-HT2A), GABAA receptor α2 (GABAARα2), GABAA receptor α3 (GABAARα3), glutamate decarboxylase (GAD) 65/67, IL-6 and IL-1β in hypothalamus and hippocampus were regulated. | (Shen et al., 2020) |
|  | Jujuboside | Male ICR mice | 9 mg/kg | Regulating 5-hydroxytryptamine energy system. | (Cao et al., 2010) |
|  | Extract of ZSS | SD rats | 13.50, 9.01 and 4.50 mg/kg | Regulating the expression levels of GABA receptor subunit alpha-1 (GABAARα1) and GABA acid receptor subunit gamma-2 (GABAARγ2) receptors in the hypothalamus and hippocampus tissue sections. | (Xiao et al., 2022) |
|  | Total saponins | SD rats | 10、20 g/kg | Enhance sleep through the 5-hydroxytryptamine mechanism. | (Zhai et al., 2015) |
| Antidepressant effect | Extract of ZSS | Rats | -- | Regulate the level of pyroglutamic acid to resist depression. | (Du et al., 2024) |
|  | Alkaloids | Male ICR mice | 15 mg/kg | The contents of norepinephrine (NE), dopamine (DA) and serotonin (5-HT) were up-regulated. | (Li et al., 2022) |
|  | Saponins | Male ICR mice | 110 mg/kg | The contents of norepinephrine (NE), dopamine (DA) and serotonin (5-HT) were up-regulated. | (Li et al., 2022) |
| Anxiolytic effect | Ethanol extract of ZS | SD rats | 60, 180 mg/kg | Improving the transmission of CRF/CRFR1 and N/OFQ/NOP in CeA. | (Li et al., 2019) |
|  | Spinosin | Mice | 2.5, 5 mg/kg | Regulates GABAA and 5-HT1A receptors | (Liu et al., 2015) |
|  | Jujuboside A | Male Kunming mice | 0.02, 0.2 mg/kg | Aβ1-42-induced histopathological damage was antagonized, the activity of caspase-9 and caspase-3 in mitochondria was decreased, and apoptosis was reduced. | (Liu et al., 2014) |
| Anti-stress ulcer effect | ZSS extract | Kunming mice | 2.5, 10 and 25 g/kg | The process of balancing central excitation and inhibition. | (Li and Zheng, 2003) |

**Supplementary Table 5.** Effects of ZSS on the cardiovascular system.

| Pharmacological  effects | Extracts/Metabolites | Model | Dose range tested | Mechanism | Ref. |
| --- | --- | --- | --- | --- | --- |
| Cardiovascular effect | Jujuboside A | Mice | 10, 20 mg/kg | -- | (Zhang et al., 2005) |
|  | Spinosa | Wistar rats | 0.1, 0.3 and 1.0 g/kg | Slowing down the heart rate to reduce the myocardium, oxygen consumption to reduce the damage of hypoxia to the myocardium ; regulate the body 's neuro-endocrine-immune system function to maintain internal environment stability ; improve myocardial blood supply ; recovery of myocardial contractility recovery | (Zhang et al., 2005) |
| Lipid-regulating effect | Saponin | SD rats | 120、240 and 480 mg/kg | The content of TC and TLDL-C in serum of hyperlipidemia animal model was decreased, the content of HDL-C was increased, and the ratio of TC/HDL was decreased. | (Chen et al., 1990) |
| Antihypertensive effect | Saponin | Rats | 5, 10 and 25 mg/kg | -- | (Wu et al., 1991) |

**Supplementary Table 6.** Effects of ZSS on the immune system.

| Pharmacological  effects | Extracts/Metabolites | Model | Dose range tested | Mechanism | Ref. |
| --- | --- | --- | --- | --- | --- |
| Anti-inflammatory effects | Jujuboside F | RAW 264.7 | 10 mM | Inhibits the release of pro-inflammatory cytokine TNF-α | (Fu et al., 2016) |
|  | Jujuboside G | RAW 264.7 | 10 mM | Inhibits the release of pro-inflammatory cytokine TNF-α | (Fu et al., 2016) |
|  | Jujuboside H | RAW 264.7 | 10 mM | Inhibits the release of pro-inflammatory cytokine TNF-α | (Fu et al., 2016) |
|  | Polysaccharides | Caco2 cells | 200, 100, 50, 25 and 12.5 μg/mL | Regulating the assembly of tight junctions involves AMPK activation | (Yue et al., 2015) |
|  | Polysaccharides | RAW 264.7 | 200, 100, 50, 25 and 12.5 μg/mL | Regulating the assembly of tight junctions involves AMPK activation | (Yue et al., 2015) |
|  | Polysaccharides | Male SD rats | 20, 40 and 80 mg/kg | Regulating the assembly of tight junctions involves AMPK activation | (Yue et al., 2015) |
| Anti-tumor effect | Jujuboside B | AGS human gastric cancer cells | 25, 50, 100 and 150 μM | Increase the activation of FasL and caspase-8. Activation of p38/c-Jun N-terminal kinase (JNK). Inhibition of exogenous pathway-mediated apoptosis by inducing protective autophagy. | (Xu et al., 2014) |
|  | Jujuboside B | HCT 116 human colon cancer cells | 25, 50, 100 and 150 μM | Increase the activation of FasL and caspase-8. Activation of p38/c-Jun N-terminal kinase (JNK). Inhibition of exogenous pathway-mediated apoptosis by inducing protective autophagy. | (Xu et al., 2014) |
|  | Oil | Mice | 1.4, 0.35 mL/kg | -- | (Wang et al., 1995) |
| Immunoregulation effect | Protein | ICR mice | 100 , 200 and 400 mg/kg | Activation of MAPKs and NF-κB signaling pathways | (Zhang et al., 2023a) |

**Supplementary Table 7.** Other pharmacological effects of ZSS.

| Pharmacological  effects | Extracts/Metabolites | Model | Dose range tested | Mechanism | Ref. |
| --- | --- | --- | --- | --- | --- |
| Antioxidation effect | 6'"-p-Coumaroylspinosin | PC12 cells | 20, 50, 100, 200, 300, and 400 nM | Inhibition of acrylamide induced Bax and Bim expression and JNKs pathway. | (Li et al., 2020) |
|  | 6'''-O-Acetylsespinorolactone | Caenorhabditis elegans | 10, 100 and 200 µM | Reduce the level of ROS and MDA, enhance the activity of GSH-Px, increase the expression of SOD-3 and GST-4, and improve the ability of antioxidant damage. | (Zhang et al., 2023b) |
| Hepatoprotective effect | Jujuboside A | Mice | 5, 10 and 20 mg/kg | Inhibition of YY1/CYP2E1 signaling alleviates T2DM-related NAFLD by activating PPARα. | (Zhang et al., 2024) |
|  | 6'''-O-Acetylsespinorolactone | HepG2 cells | 1.25, 2.5 and 5 μM | Inhibition of YY1/CYP2E1 signaling alleviates T2DM-related NAFLD by activating PPARα. | (Wang et al., 2024) |
